# Supplementary material for: Systems acclimation to osmotic stress in zygnematophyte cells
Source: Nat Commun. 2026 Jan 16;17:755. doi: 10.1038/s41467-026-68329-z (PMC12820192; doi:10.1038/s41467-026-68329-z)

Supplementary Figure(s) 1. new protein groups

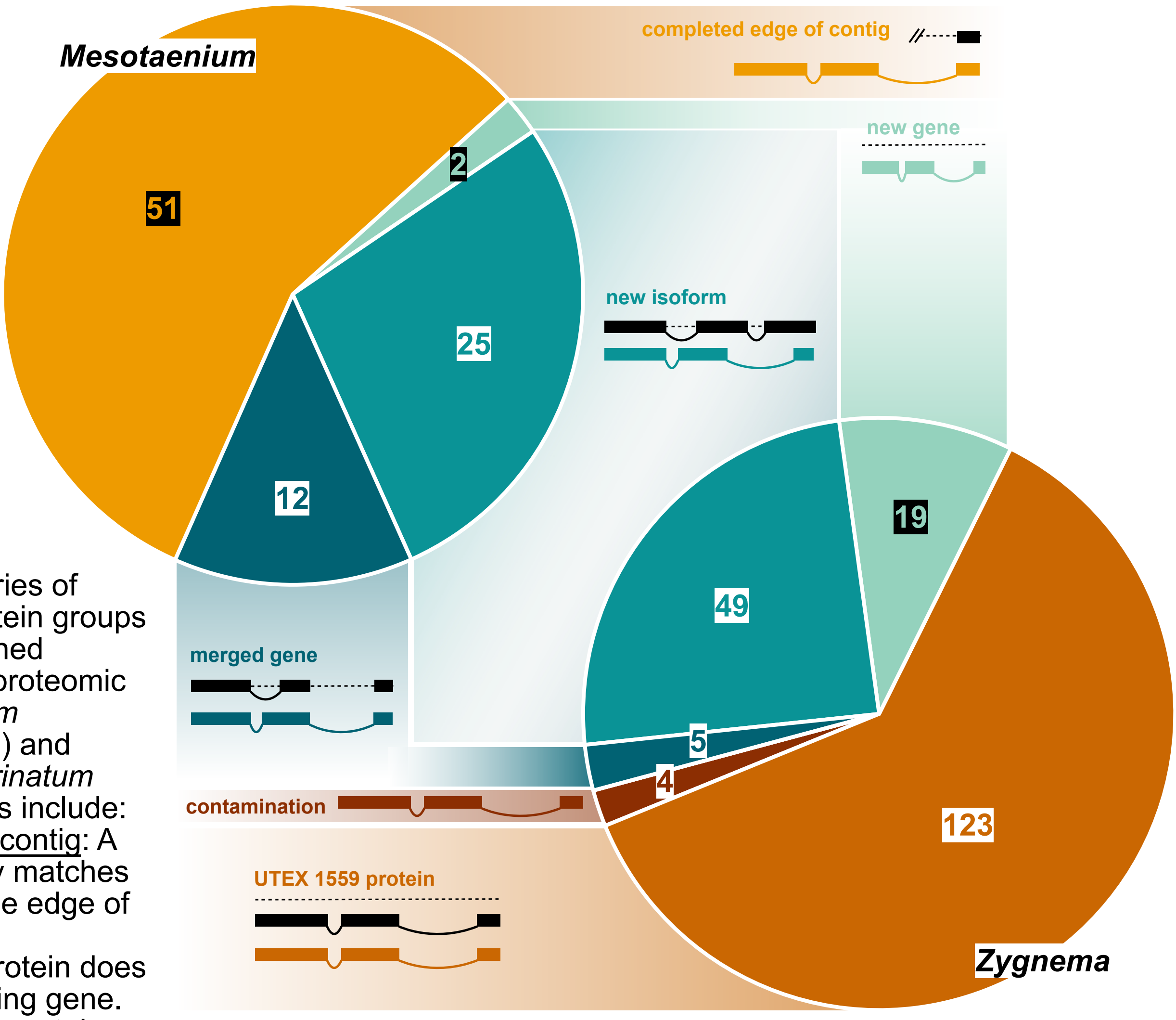

Figure 1.

Top Panel: Categories of newly identified protein groups derived from combined transcriptomic and proteomic data of *Mesotaenium endlicherianum* (Me) and *Zygnema circumcarinatum* (Zci). The categories include:
Completed edge of contig: A new protein partially matches a gene located at the edge of a contig.
New gene: A new protein does not match any existing gene.
New isoform: A new protein group matches an existing gene but represents a novel isoform.
Merged gene: A new protein spans two adjacent genes transcribed in the same direction, effectively merging them.
Contamination: A new protein originates from external contamination, not the studied algae.
UTEX 1559 protein: A new protein is not annotated as a gene in strain 698-1b but is annotated in strain UTEX 1559.

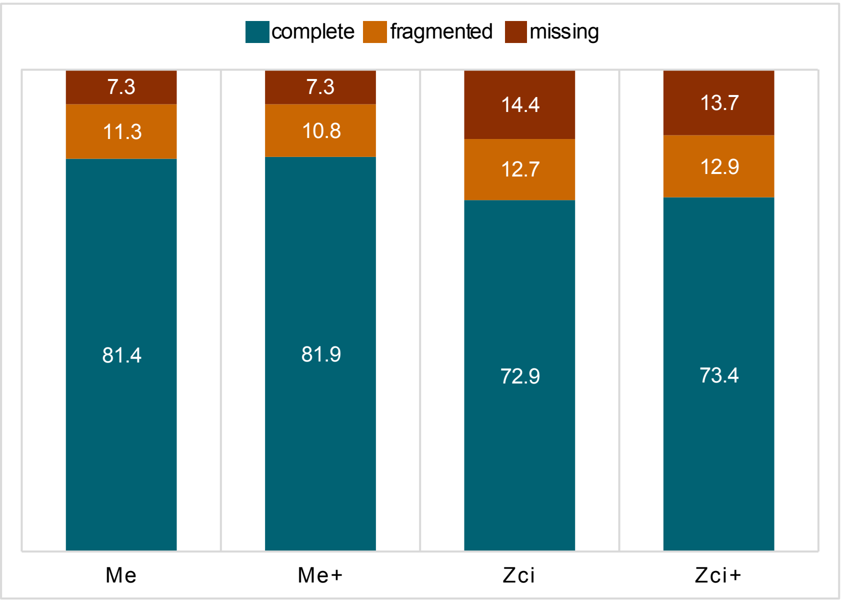

Bottom Panel: BUSCO (Benchmarking Universal Single-Copy Orthologs) scores of the proteomes for both algae, shown with (+) and without (-) the newly identified protein groups.

Supplementary Figure(s) 2. Protein-RNA correlations

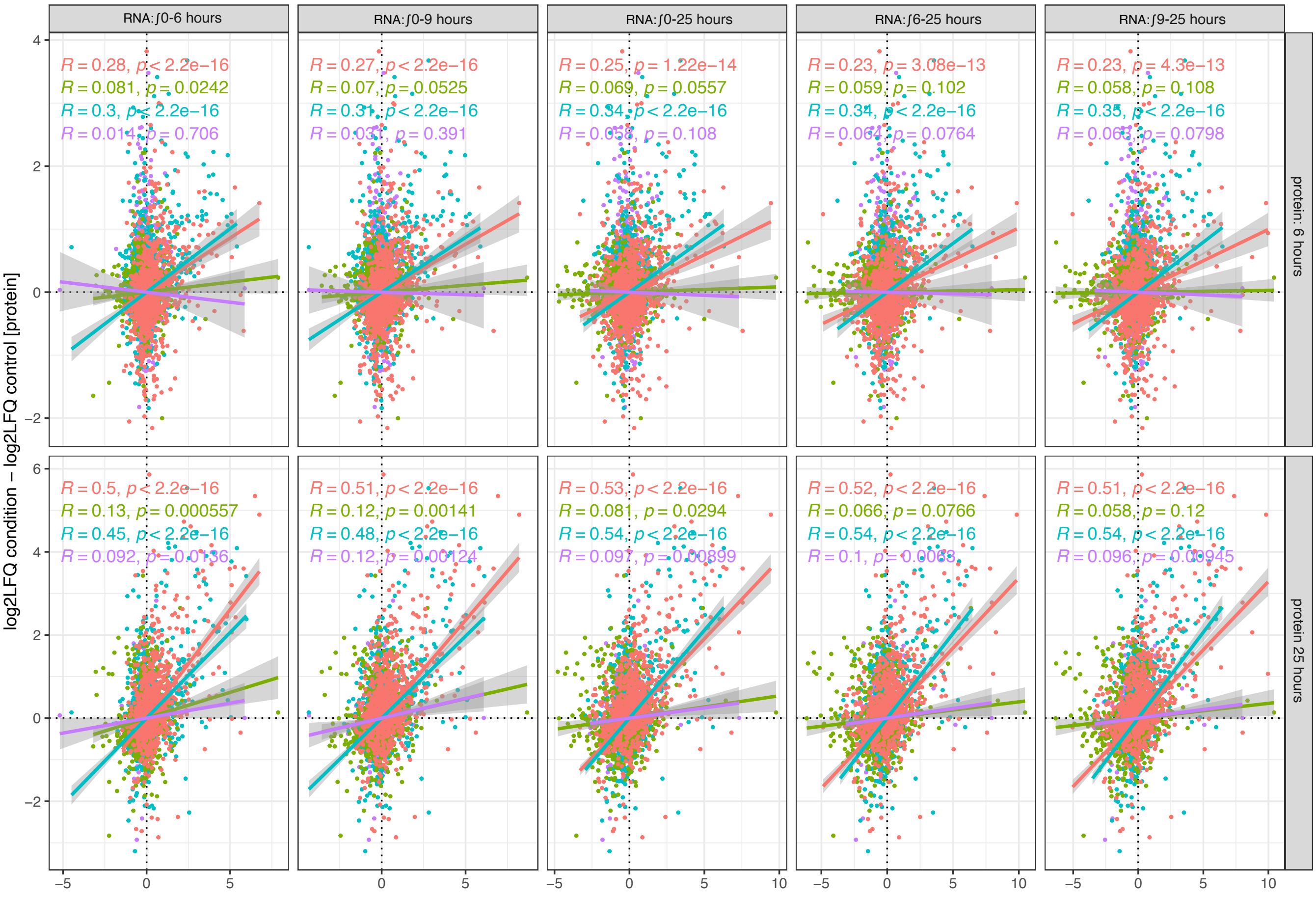

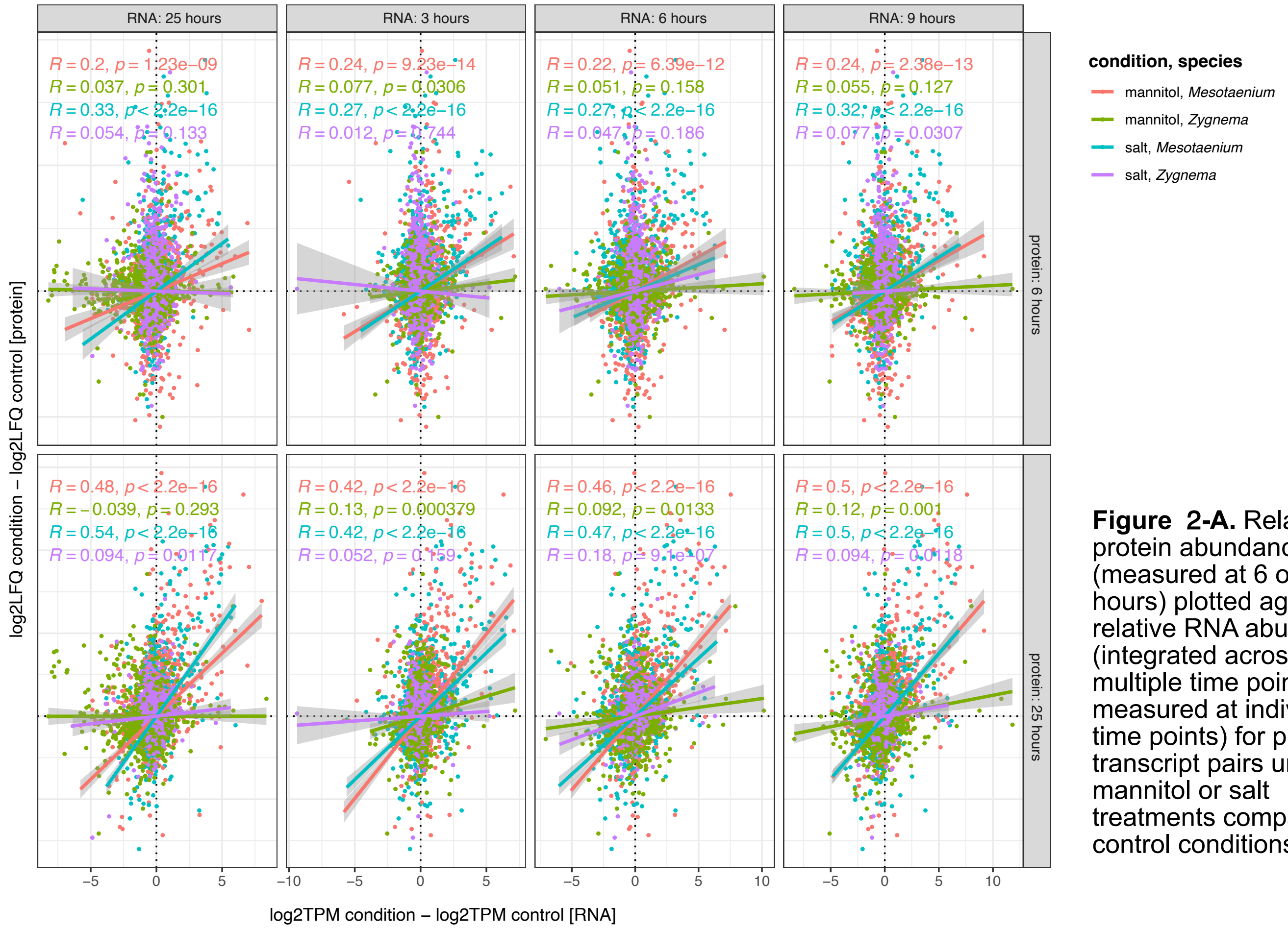

Figure 2-A. Relative protein abundance (measured at 6 or 25 hours) plotted against relative RNA abundance (integrated across multiple time points or measured at individual time points) for protein-transcript pairs under mannitol or salt treatments compared to control conditions.

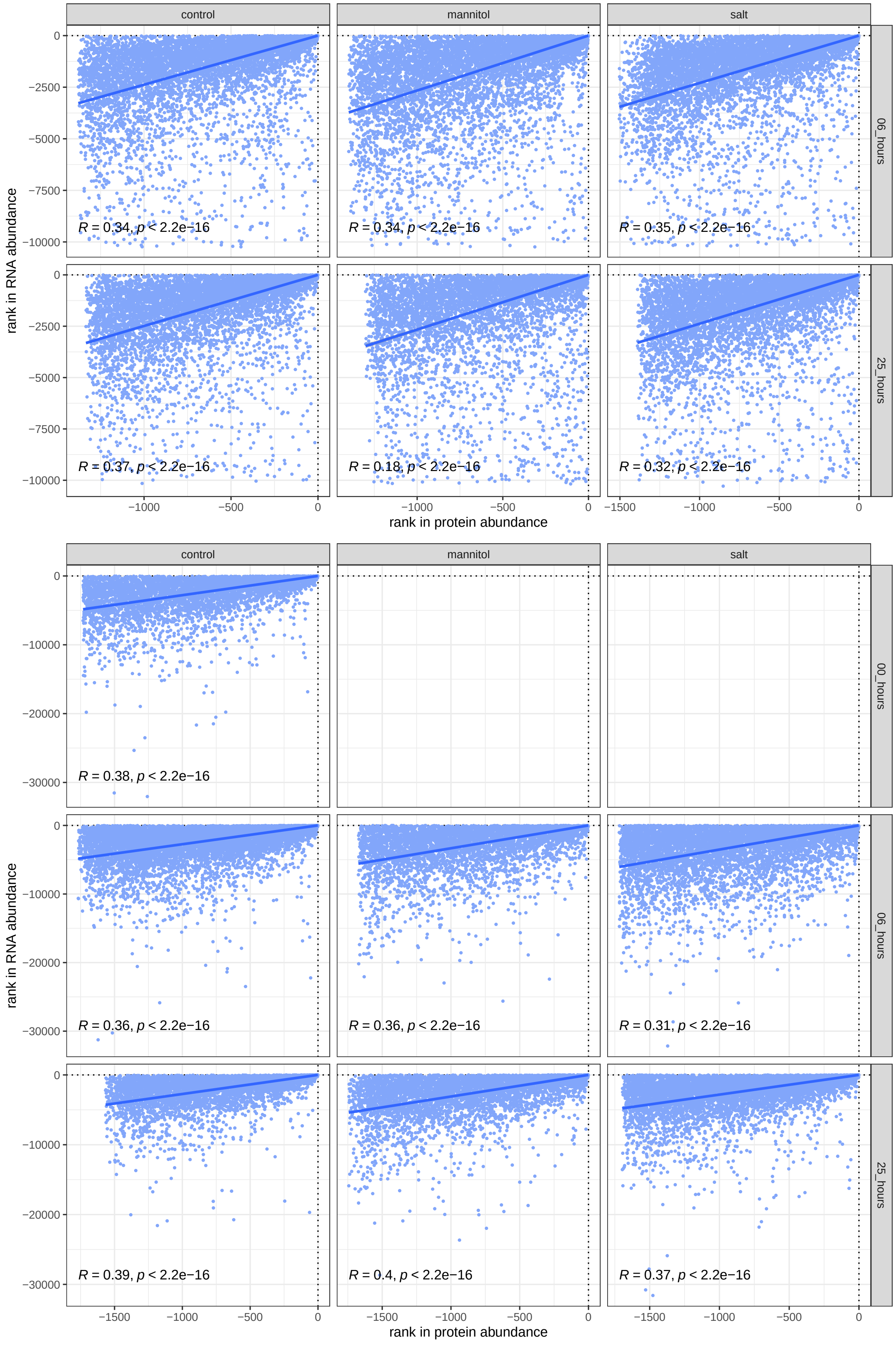

Figure 2-B. Absolute protein abundance in *Zygnema* (top graph) and *Mesotaenium* (bottom graph) plotted against absolute RNA levels.

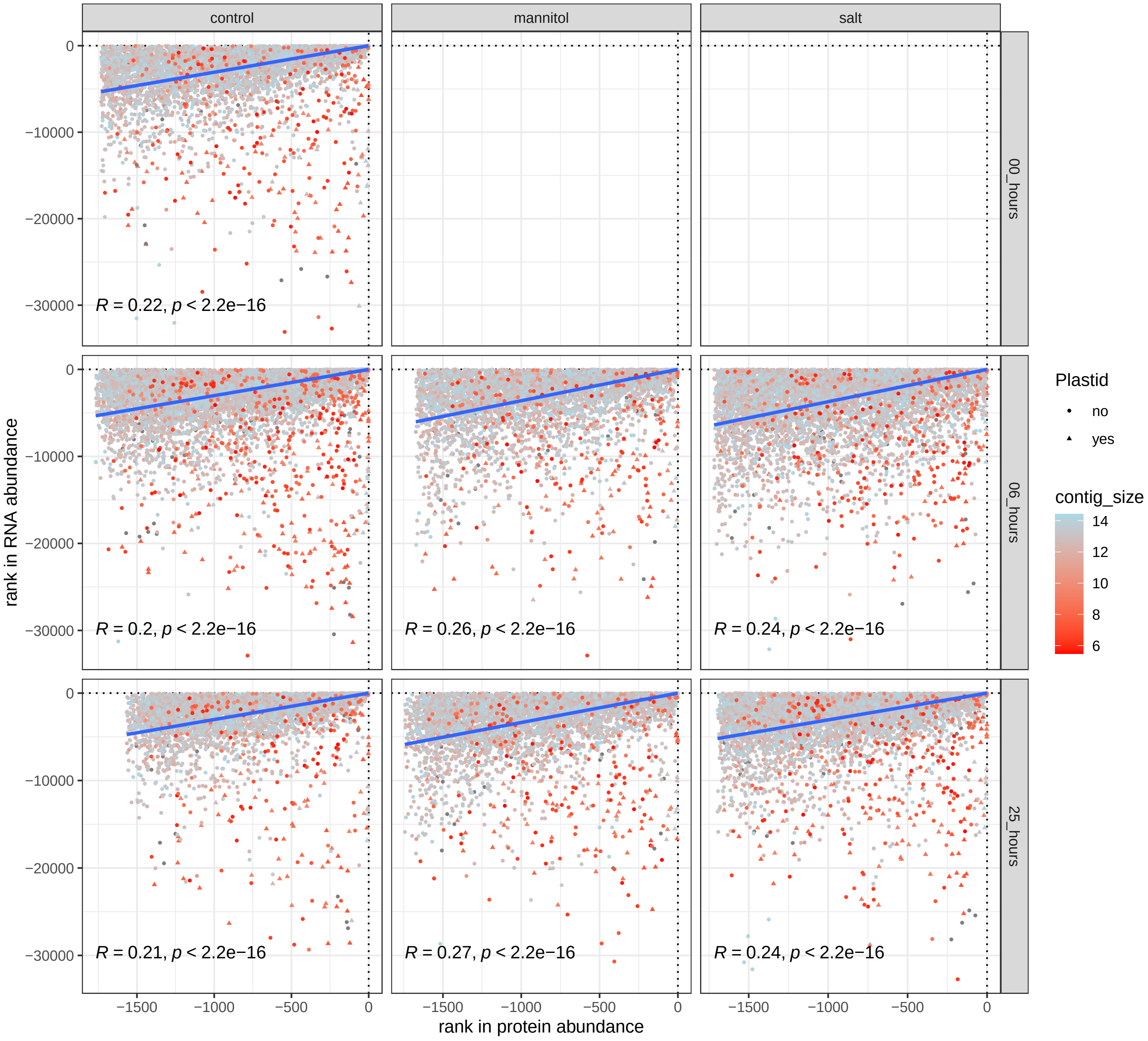

Figure 2-C. Absolute protein abundance in Mesotaenium (bottom graph) plotted against absolute RNA levels, with data points color-coded based on the size of the contig containing the corresponding protein-coding gene.

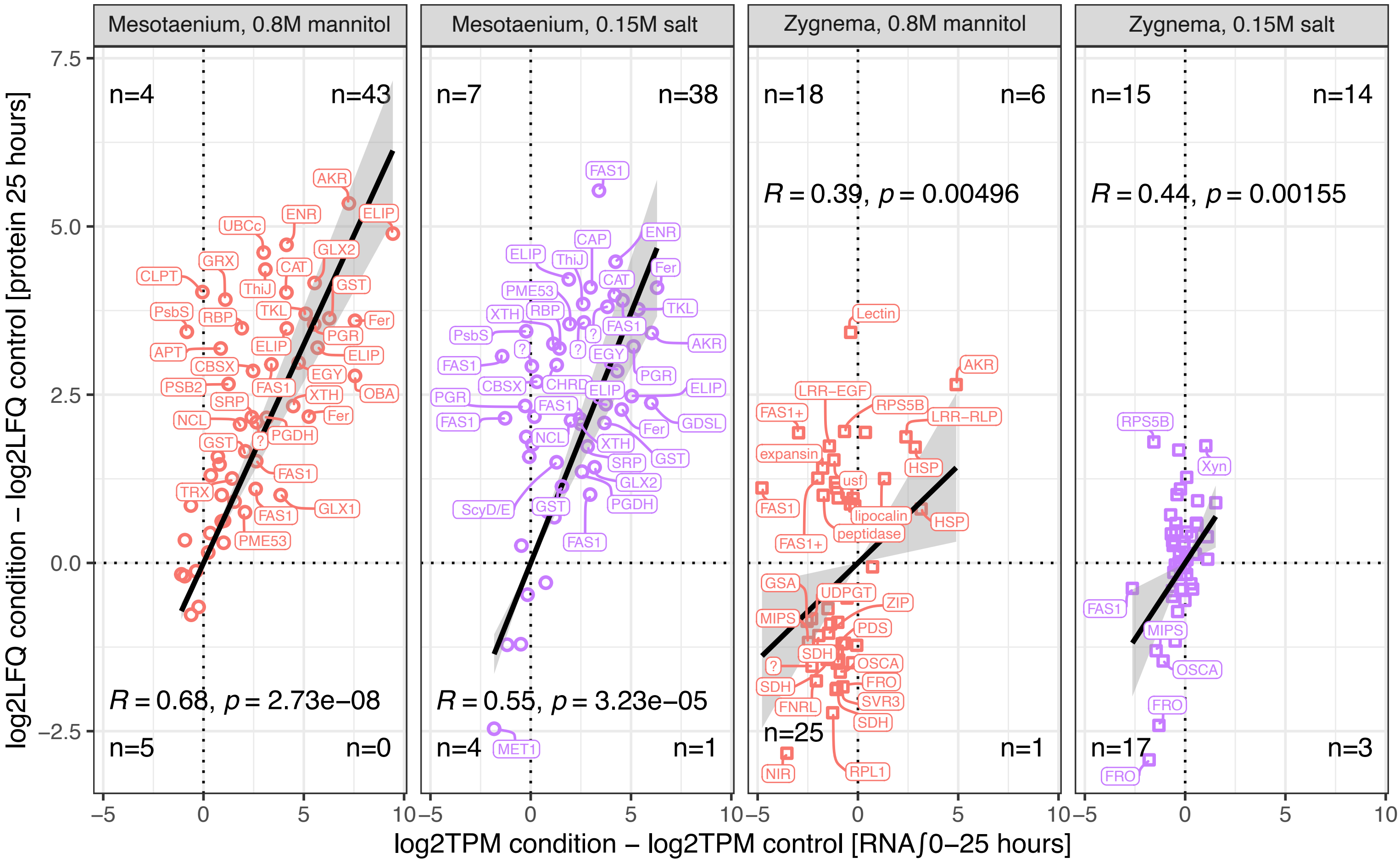

Figure 2-D.
Relative protein abundance at 25 hours is compared to relative RNA abundance (integrated from 0 to 25 hours) for proteins exhibiting a significant fold change (FDR < 0.05) under either salt or mannitol treatments relative to the control. Protein abbreviations are labeled when the summed absolute values of log2 fold changes (LFQ and TPM) exceed 2.5. Full protein names are provided in Supplementary Data II.

Supplementary Figure(s) 3. SWING

Figure 3-A. Gene regulatory networks generated using Sliding Window Inference for Network Generation (SWING). The large cyan networks display the top 1,750 predicted edges, while the smaller red networks highlight the top 0.1% of predicted edges. In the cyan networks, nodes representing genes with connectivity greater than 12 are labeled with their corresponding transcript.

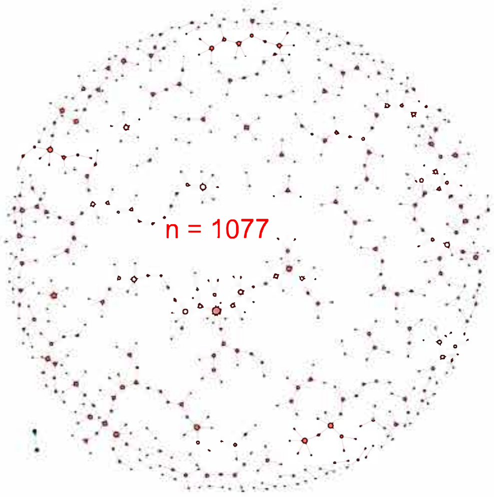

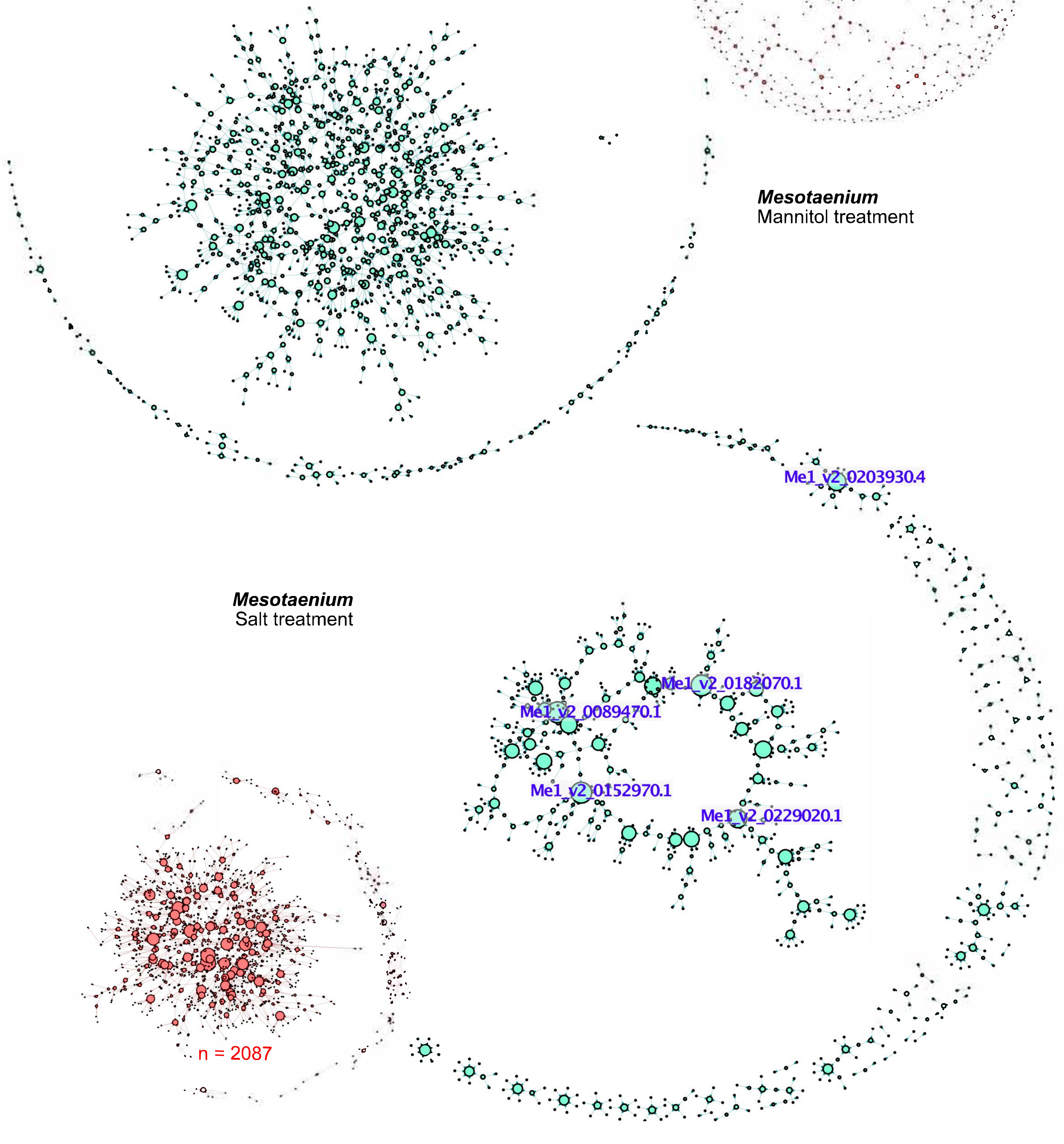

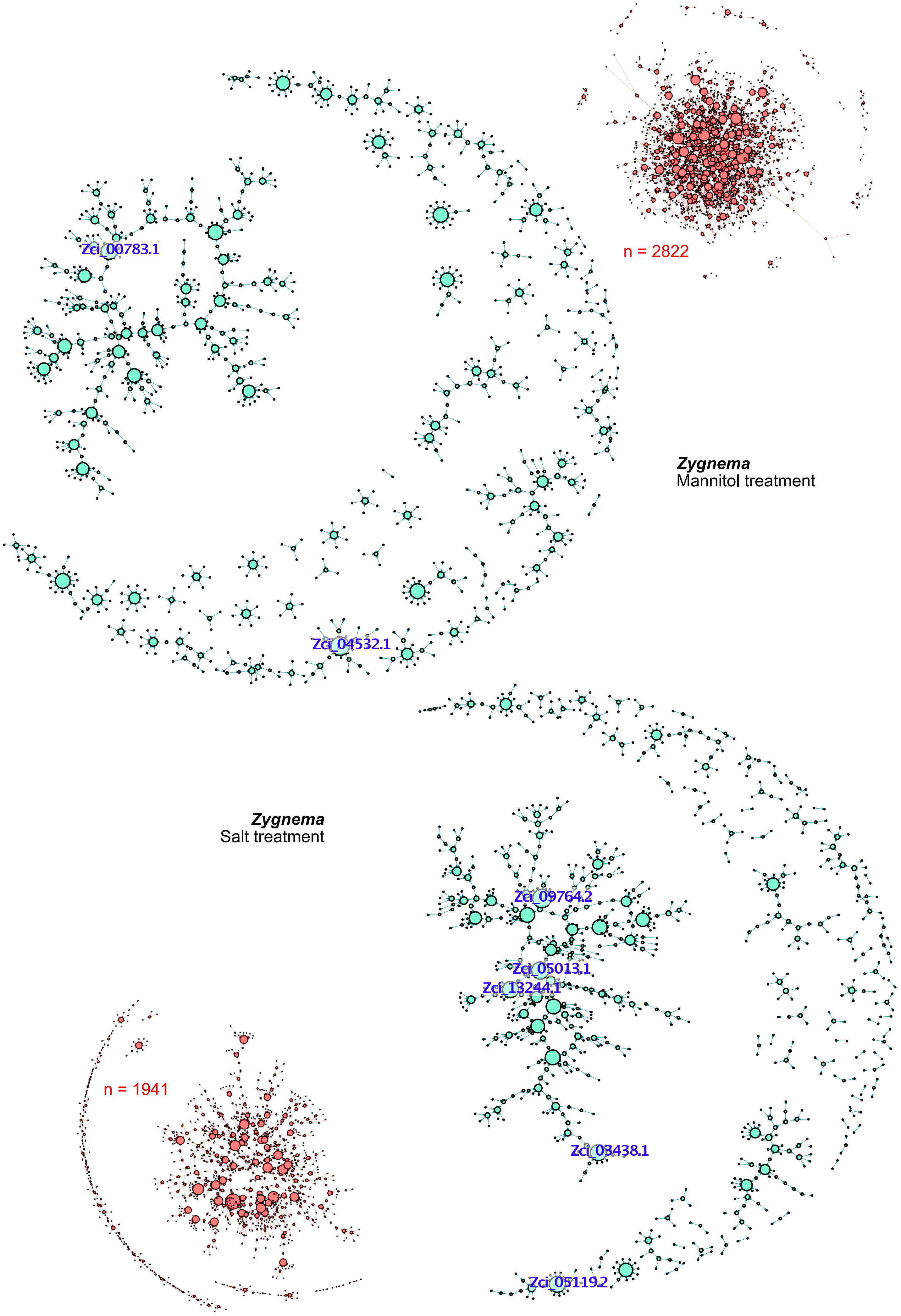
n = 2822
Zci_00783.1
Zci_04532.1
Zygnema
Mannitol treatment
Zygnema
Salt treatment
Zci_09764.2
Zci_05013.1
Zci_13244.1
Zci_03438.1
Zci_05119.2
n = 1941

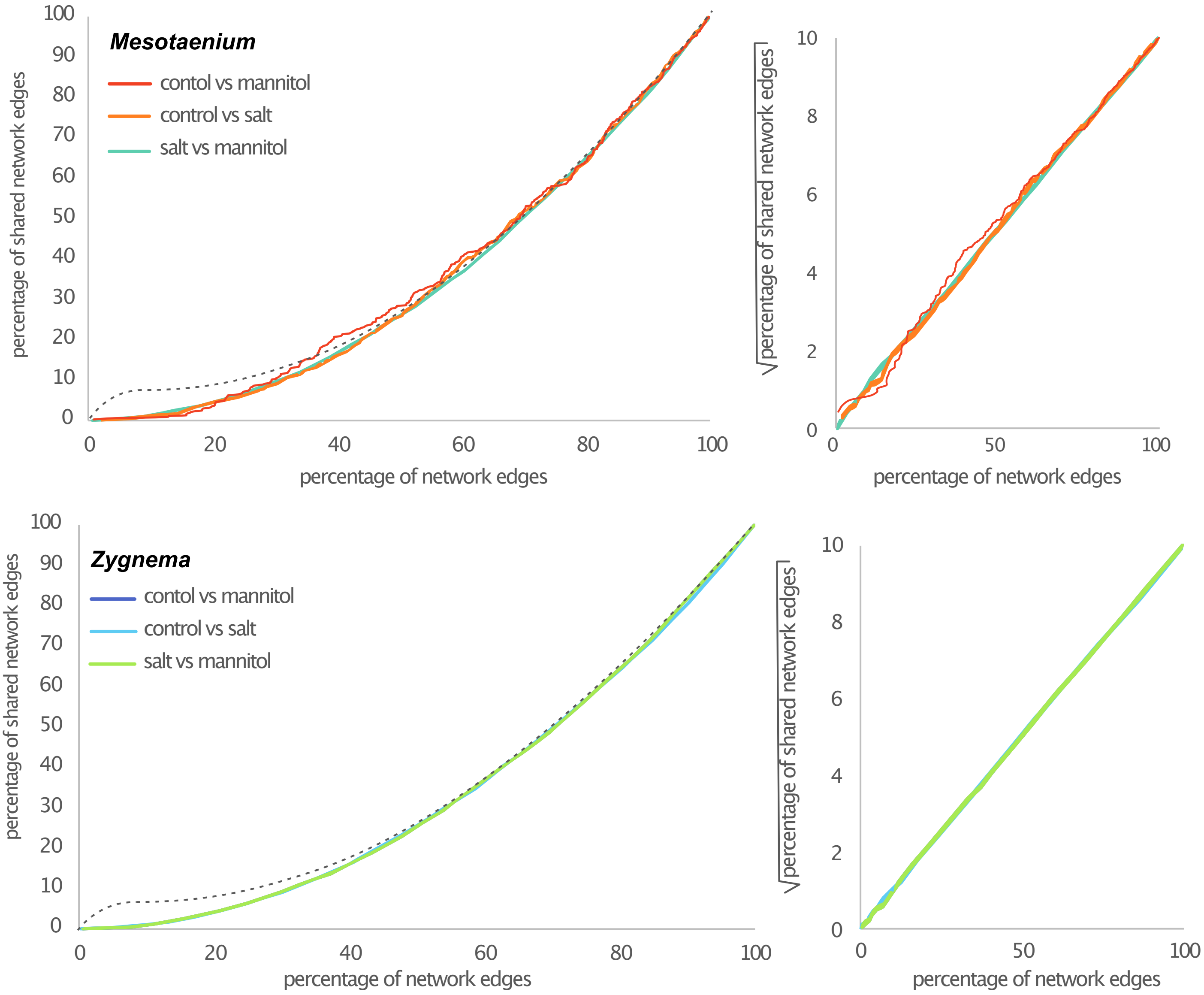

Figure 3-B. The X-axis represents the percentage of all shared network edges (between two treatments), ranked by score from highest to lowest. The Y-axis shows the percentage of those edges, compared to the total amount of shared edges, that are identical between the two treatments. A steep initial slope in the curve would indicate a higher similarity in the gene regulatory networks between two treatments (as examplified with the dotted line), while the observed trend may suggest uncorrelated gene regulatory networks.

Supplementary Figure(s) 4. expression patterns

Figure 4. Expression levels (transcripts per million, TPM) for selected genes are presented. Data represent two species: *Zygnema* (Zci) and *Mesotaenium* (Me). Measurements were taken at five time points: 0 hours, 3 hours, 6 hours, 9 hours, and 25 hours.

treatment
- control
- salt
- mannitol

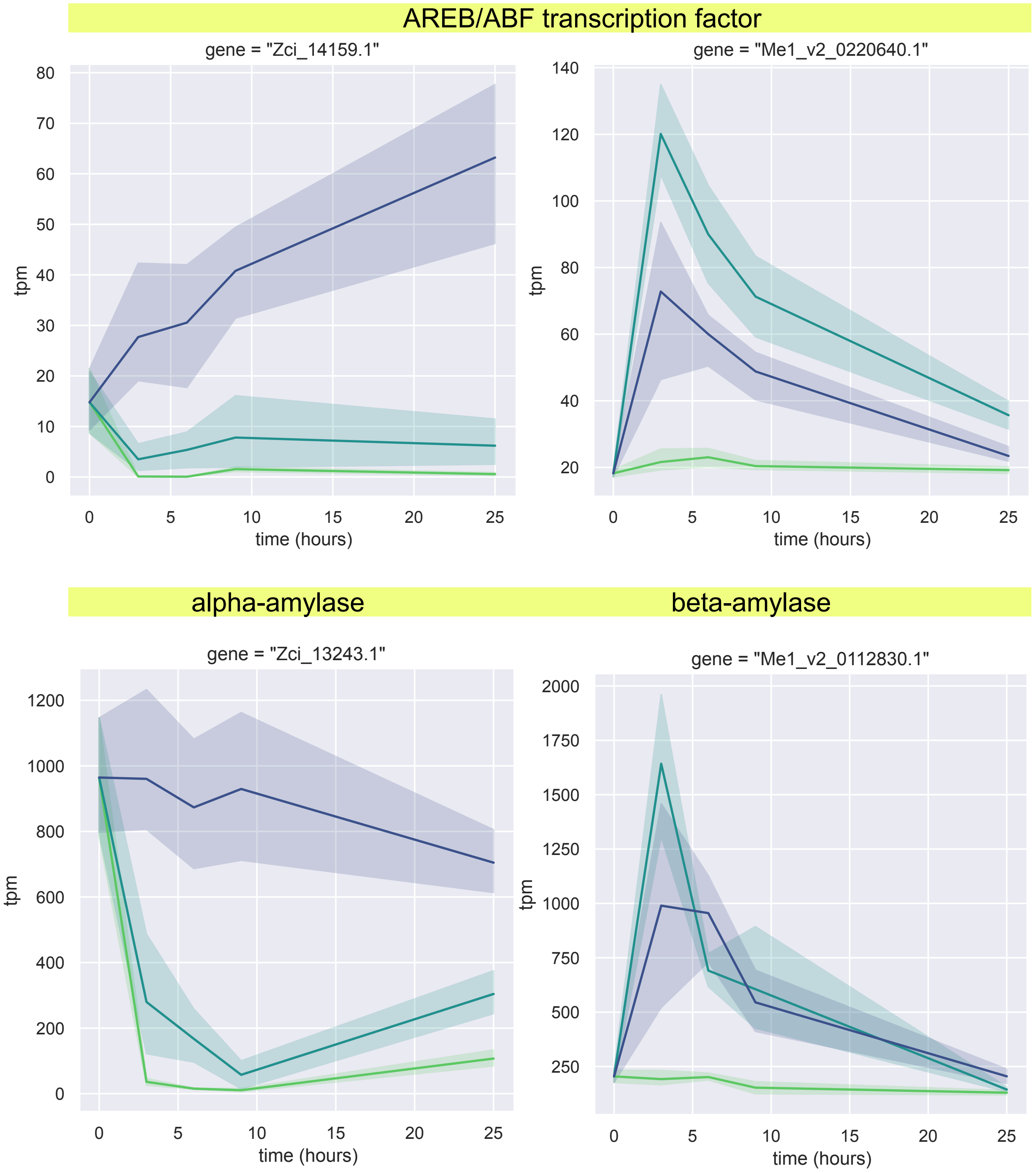

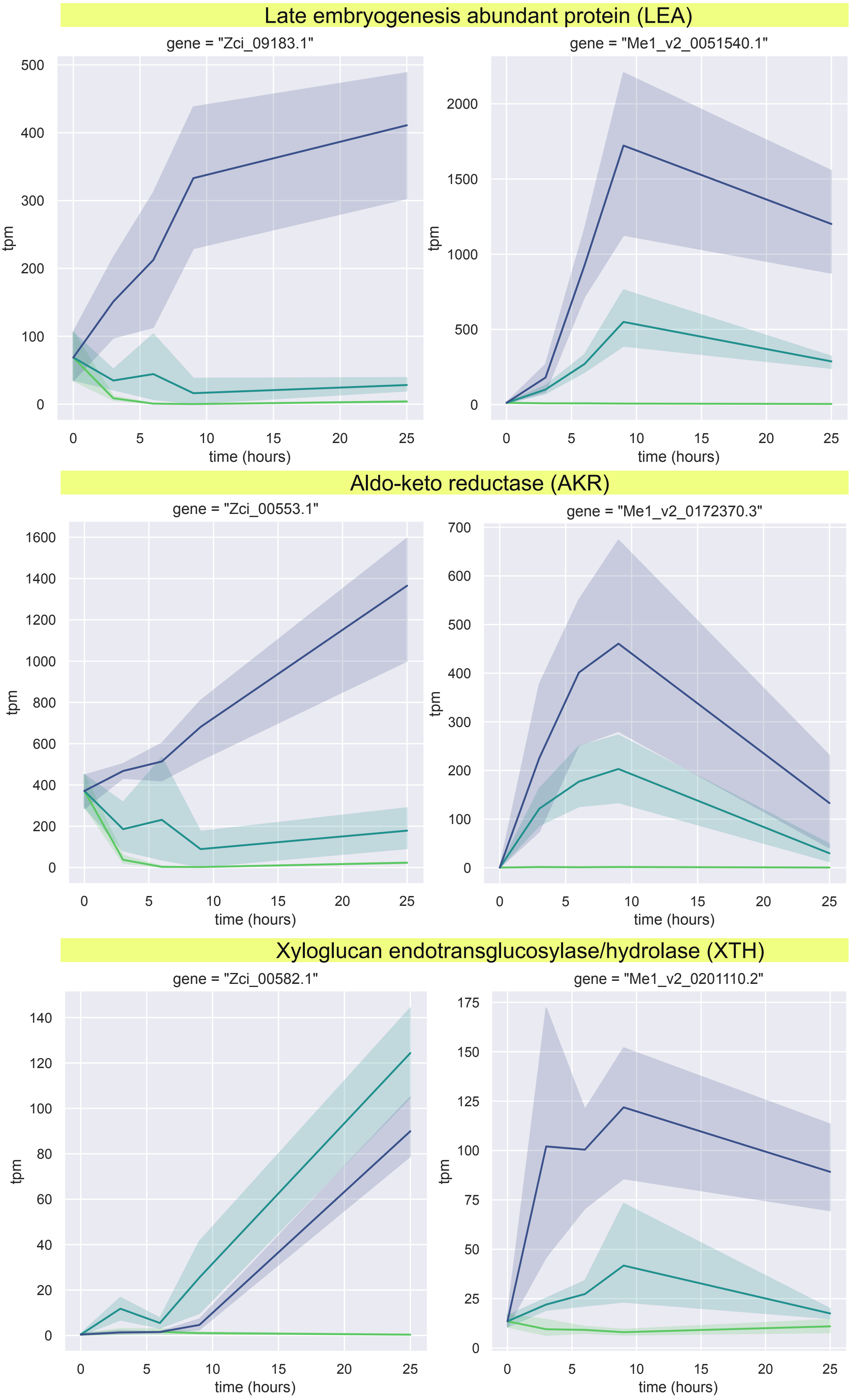
Late embryogenesis abundant protein (LEA)
gene = "Zci_09183.1"
gene = "Me1_v2_0051540.1"
Aldo-keto reductase (AKR)
gene = "Zci_00553.1"
gene = "Me1_v2_0172370.3"
Xyloglucan endotransglucosylase/hydrolase (XTH)
gene = "Zci_00582.1"
gene = "Me1_v2_0201110.2"
tpm
time (hours)

Fasciclin-Like Arabinogalactan protein (FLA)

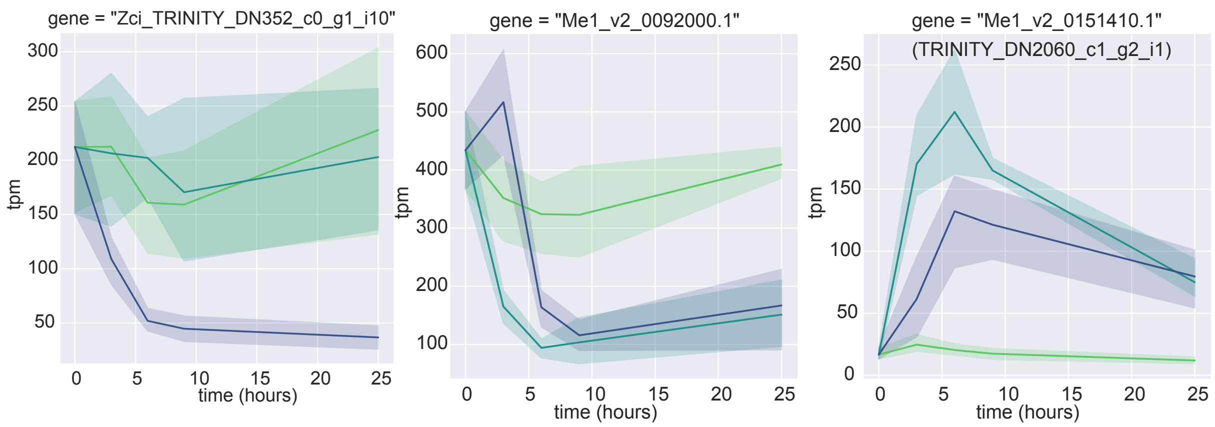

pectin methylesterase (PME)

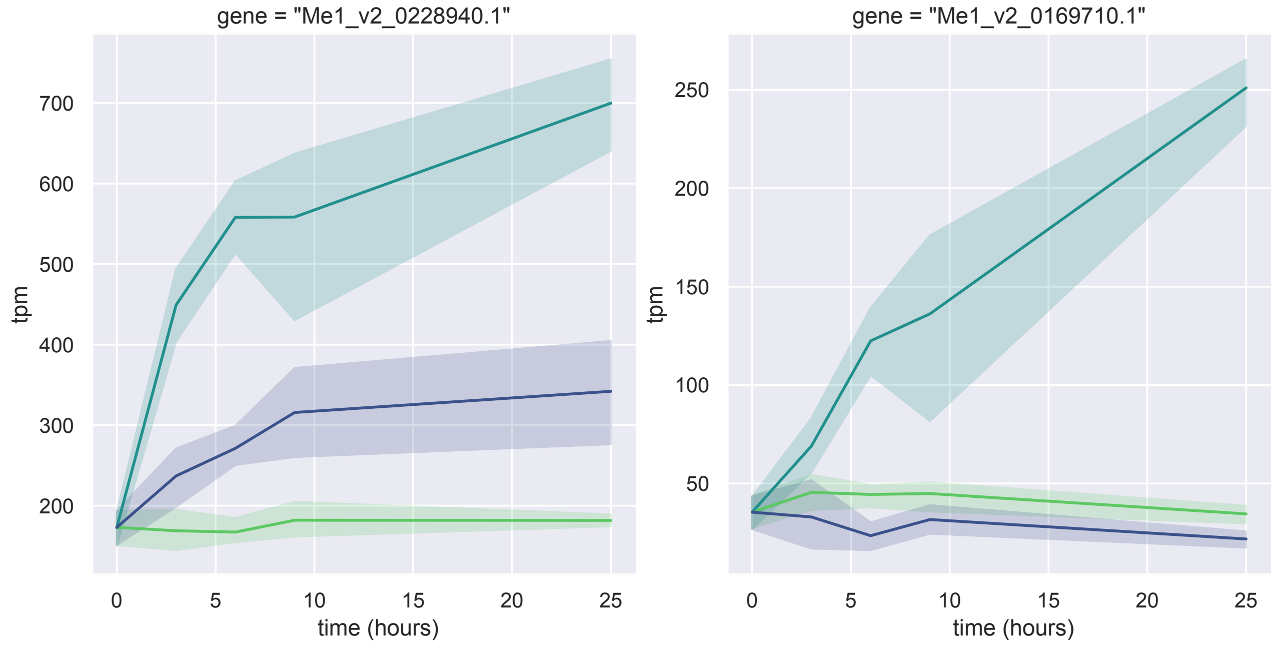

Supplementary Figure(s) 5. Metabolomics

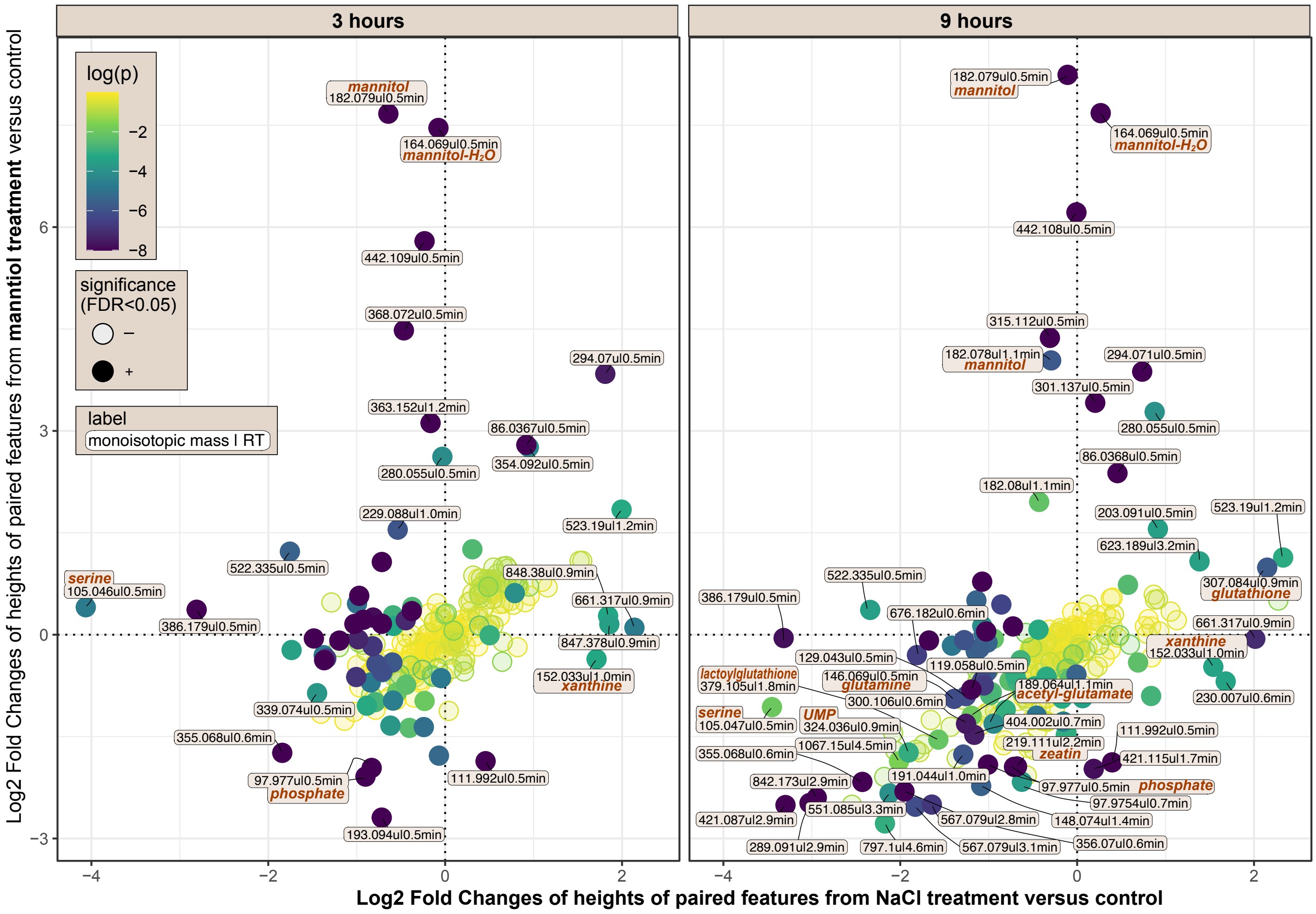

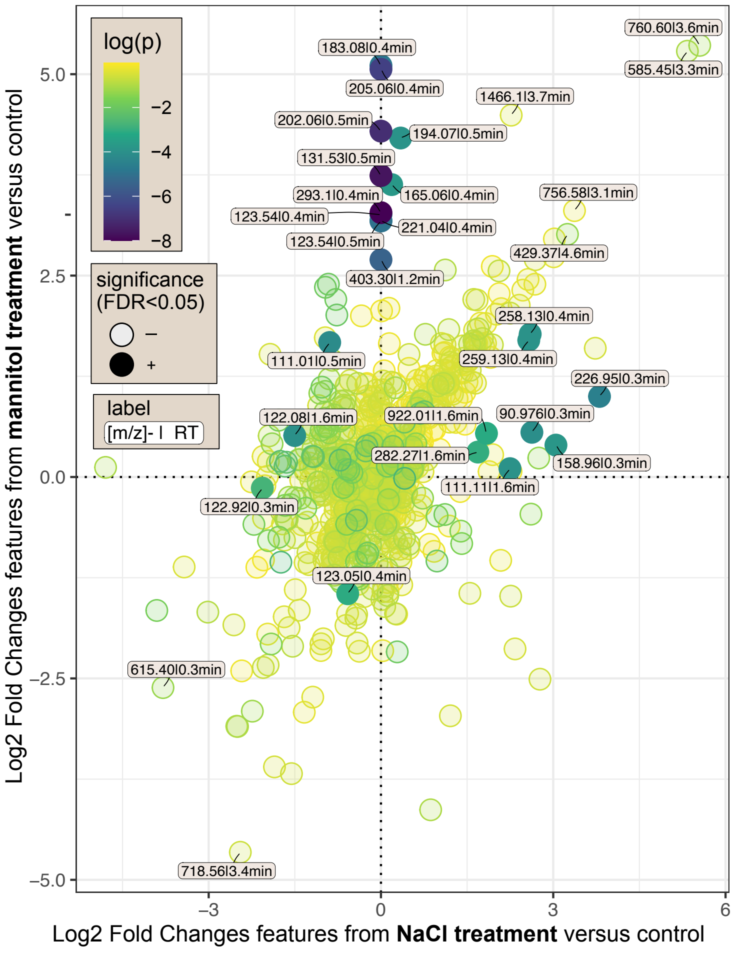

Figure 5A Comparison of the Log2 fold changes of features from LC-MS data of metabolite extracts of *Mesotaenium* subjected to osmotic treatments versus control conditions. The osmotic treatments include 0.8 M mannitol and 0.15 M NaCl. The Log2 fold changes for the 0.8 M mannitol treatment are plotted against those for the 0.15 M NaCl treatment.

Top panel) The features shown are detected in both positive and negative ion modes of methanol-based metabolite extracts. Data from the two ion modes were combined for pairs of features where the mass/charge (m/z) difference corresponds to the mass of two hydrogen atoms (2.01568 u), with a maximum allowed deviation of 0.002 u. Additionally, retention times were matched within a maximum deviation of 0.04 minutes. Unpaired features not shown. For paired features with combined absolute Log2 fold changes greater than 2 and a false discovery rate (FDR) less than 0.05, labels indicate the inferred monoisotopic weight (calculated as the average of m/z values in both ion modes) and the retention time. Where applicable, the names of putative compounds are provided based on monoisotopic weights that match entries in the BioCyc databases for *Arabidopsis thaliana*, *Klebsormidium flaccidum*, and *Chlamydomonas reinhardtii*.

Bottom) This panel presents features detected in the positive ion mode of lipid extracts from *Mesotaenium* after 9 hours of treatment. For features with an FDR less than 0.05 and/or a summed Log2 fold change exceeding |6|, labels denote the corresponding m/z values and retention times.

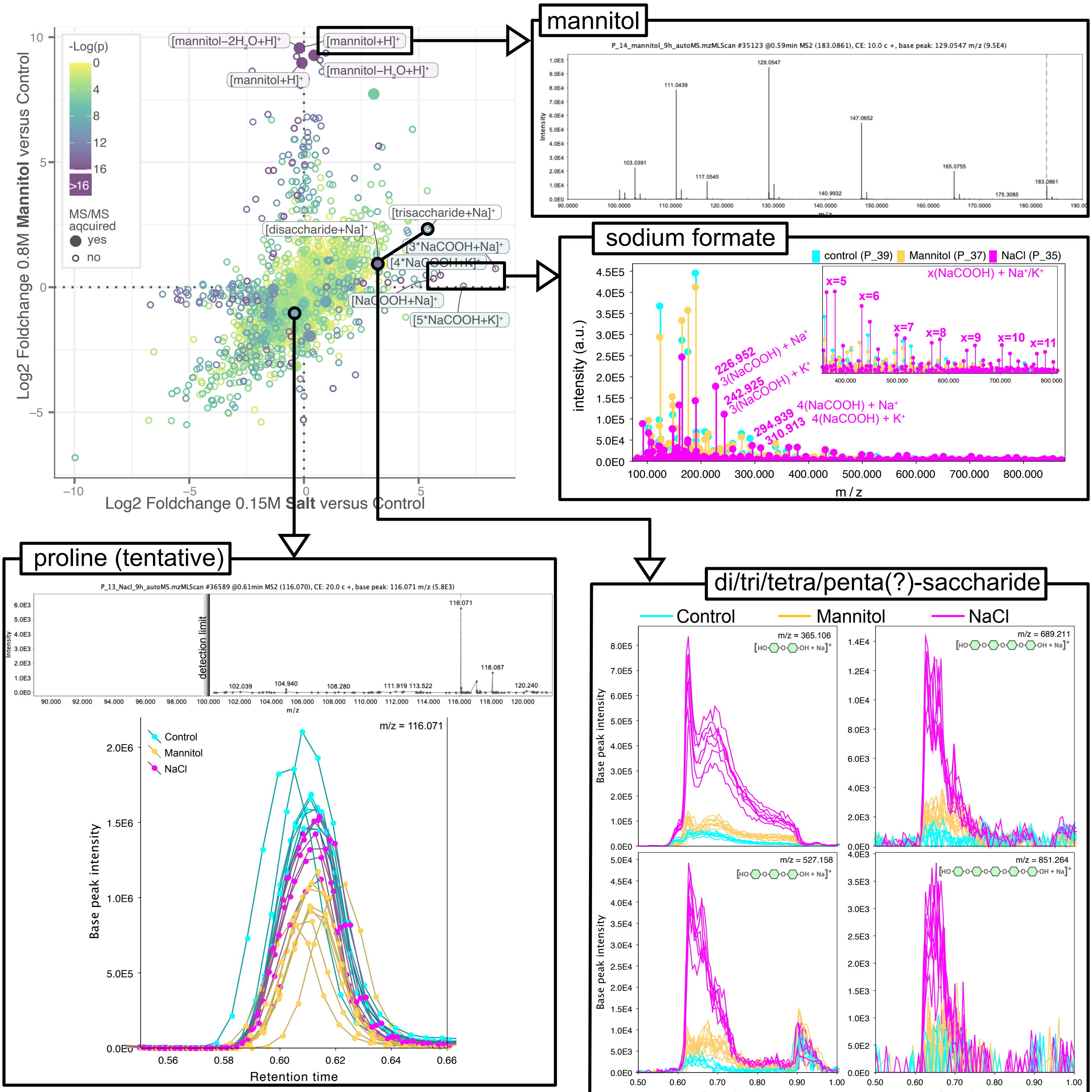

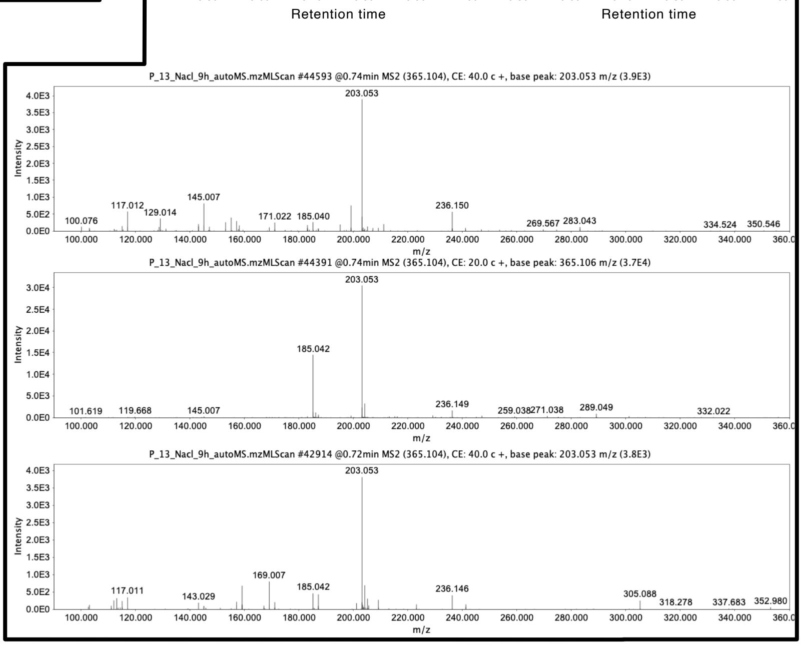

Figure 5B Chromatograms and Mass Spectra Supporting Features Visualized in Figure 6a

Mannitol: One of the 33 acquired fragmentation mass spectra tentatively identified as mannitol.

Sodium Formate: Mass spectra from a control sample (scan #28664), an NaCl-treated sample (scan #28636), and a mannitol-treated sample (scan #28638) at a retention time (RT) of 0.49 minutes.

Saccharides: Total ion chromatograms of all samples for features with RTs around 0.64 minutes and m/z values of 365.106, 527.158, 689.211, and 851.264. For the feature at m/z = 365.106, three of the nine acquired fragmentation spectra are shown.

Proline: The feature most likely corresponding to proline (based on m/z) is displayed. The top panel shows the fragmentation spectrum, and the bottom panel shows the total ion chromatograms of all samples.

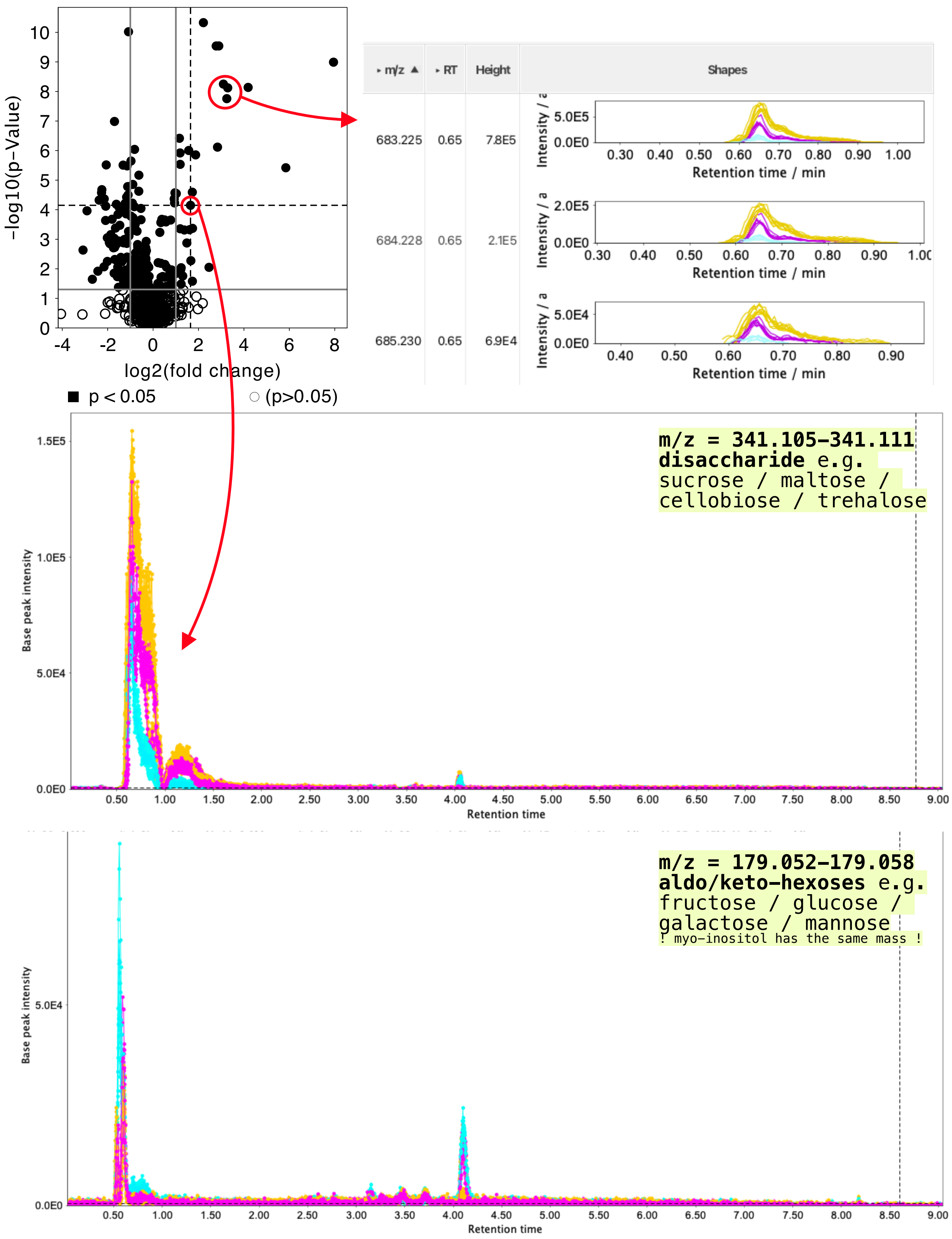

Figure 5C Top left panel: Volcano plot illustrating non-gap-filled features in negative ion mode, comparing 9-hour mannitol treatment to 9-hour control treatment of methanol-based metabolite extracts of *Mesotaenium*.
Top right panel: A feature group representing an unidentified compound that exhibits a strong increase in intensity in response to osmotic stress in Mesotaenium.
Bottom panels: Total ion chromatograms of methanol-based extracts from Mesotaenium collected after 9 hours of treatment, analyzed in negative ion mode. The chromatograms highlight two specific m/z ranges: 341.105–341.111,

corresponding to the mass of various disaccharides ($[M-H]^-$), and 179.052–179.058, corresponding to the mass of aldo- or keto-hexoses ($[M-H]^-$, e.g., glucose and fructose).
Color coding: NaCl = magenta, Control = cyan, Mannitol = orange.

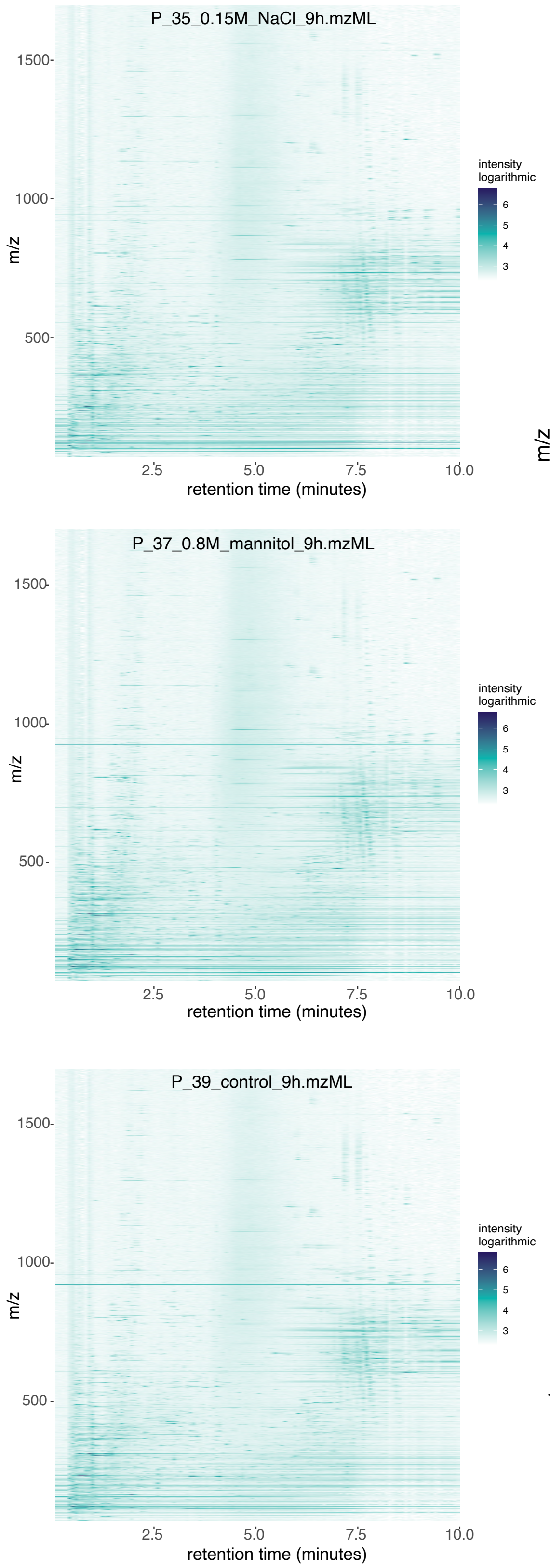

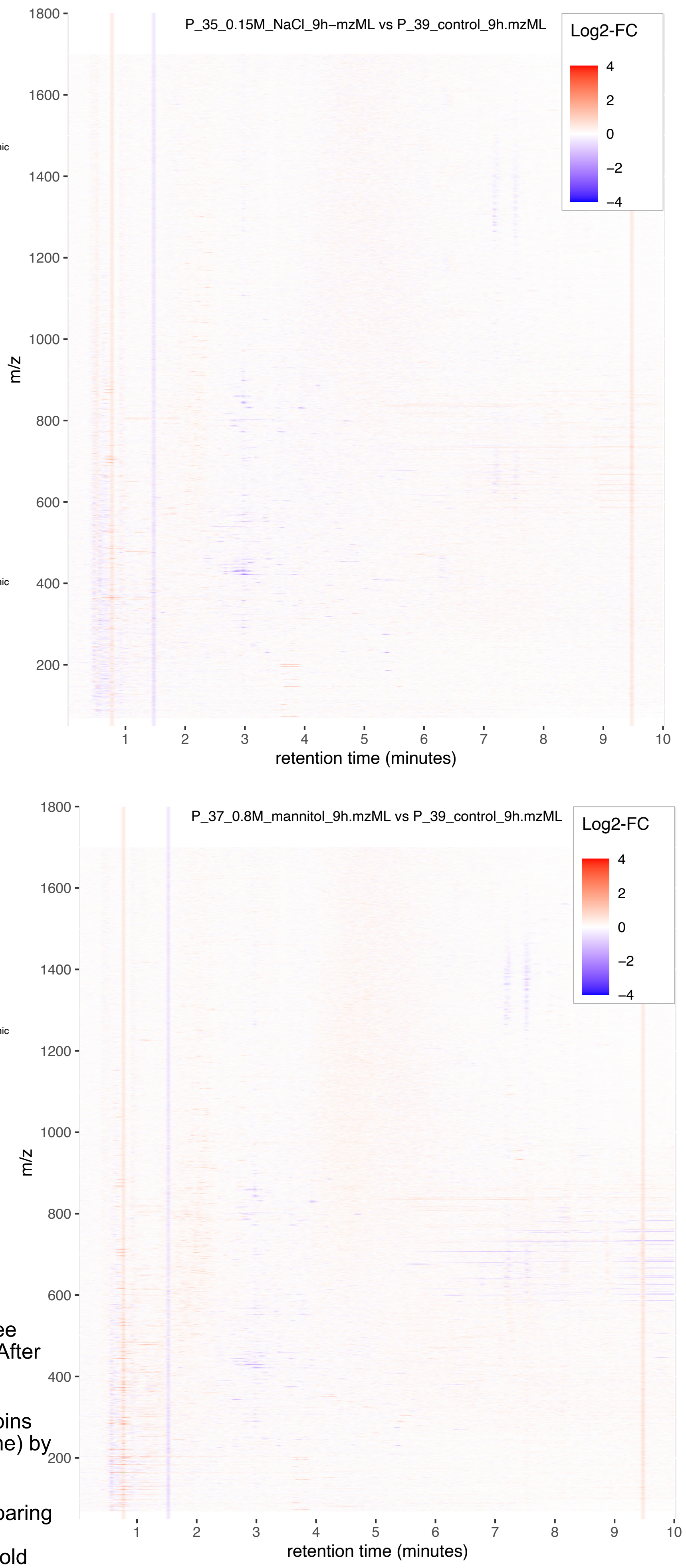

Figure 5D 2D LC-MS Chromatograms of three Methanol-Based Extracts from *Mesotaenium* After 9 Hours of Treatment (Positive Ion Mode).

Left Panel: Average signal intensities across bins with dimensions of 0.05 minutes (retention time) by 0.5 (mass-to-charge ratio).

Right Panel: Fold changes of these bins comparing samples treated with 0.15 M NaCl or 0.8 M mannitol to the control sample. For bins with fold changes exceeding |4| (only three bins in total), bins are shown in green.

Supplementary Figure 6. Sugar analysis

A. Total sugar content

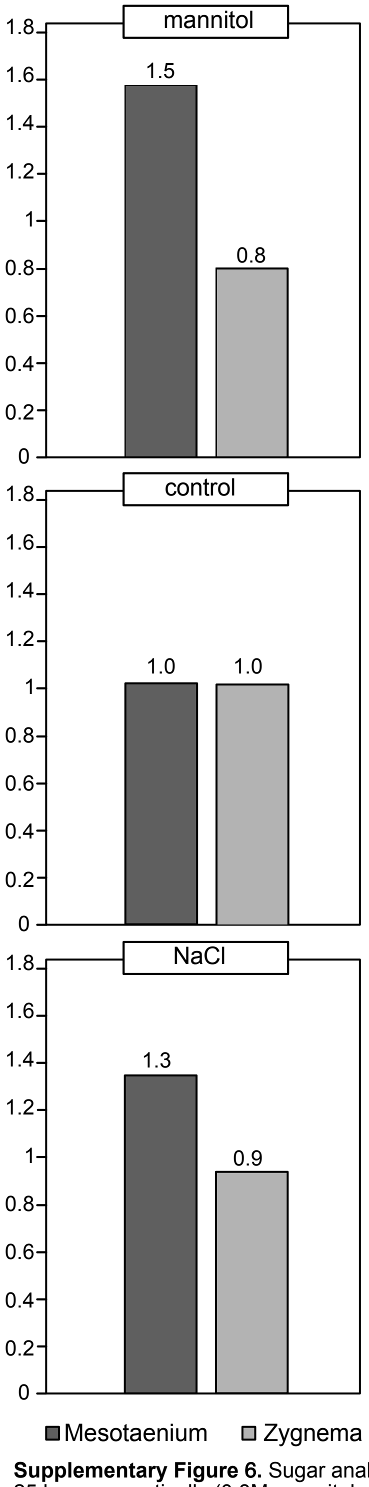

B. Uronic acid content

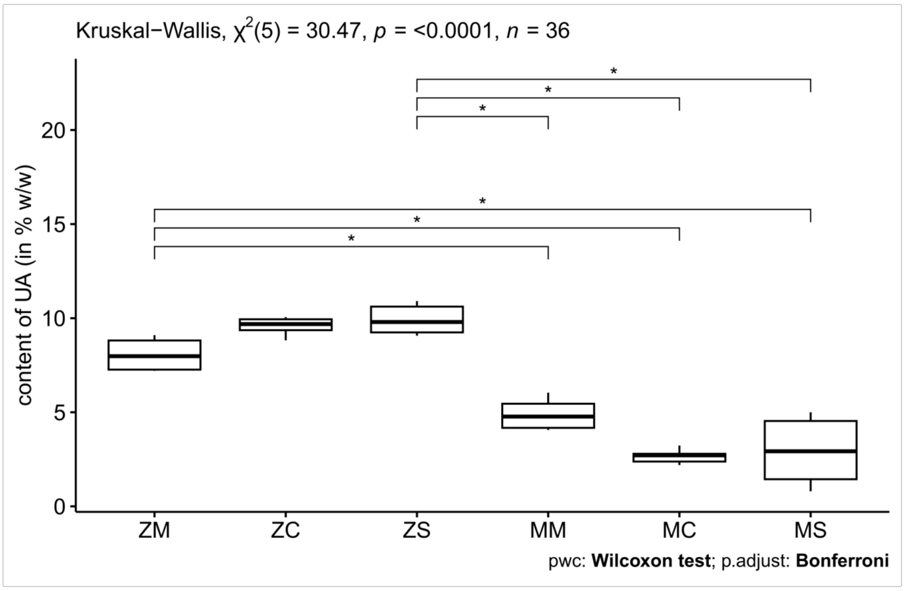

C. Hydroxyproline quantification

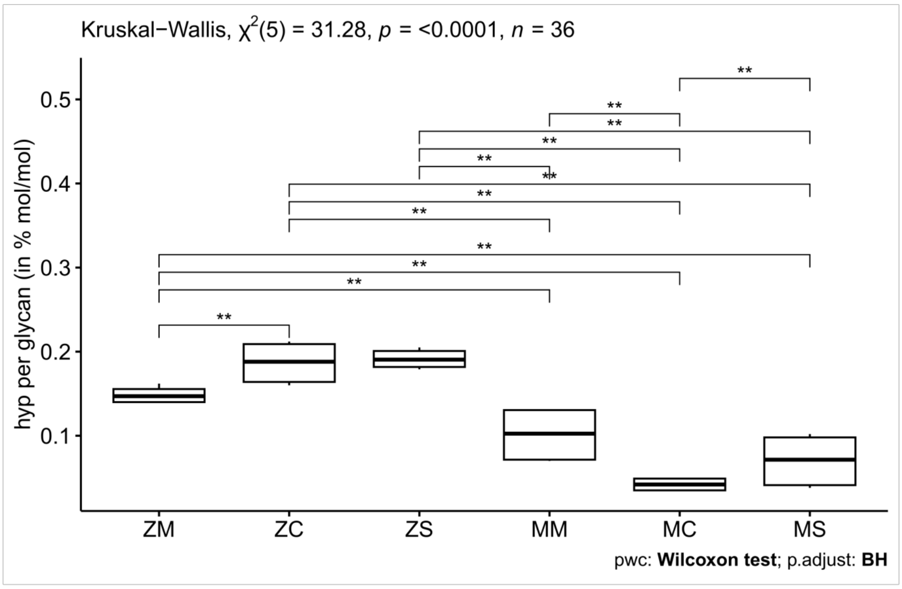

D. Neutral sugar composition

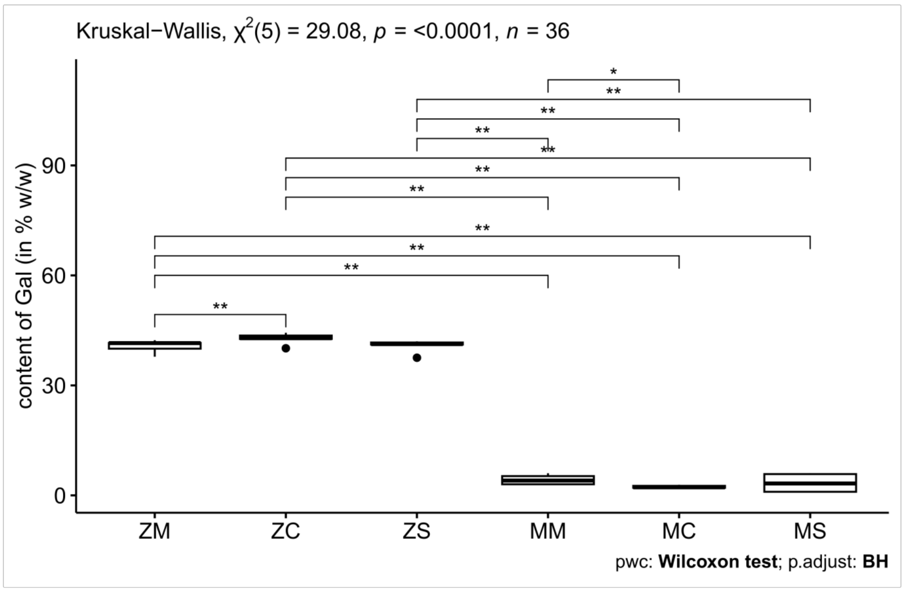

Supplementary Figure 6. Sugar analysis of 25 hours osmotically (0.8M mannitol or 0.15M NaCl) stressed algae. Averages shown from 3x technical replecate, 2x biological replicate. **A)** Total sugar content of all neutral and acidic sugars determined by colorimetric assay according to Dubios et al. (1956) **B)** Uronic acid content determined by colorimetric assay according to Blumenkrantz and Asboe-Hansen (1973). **C)** Hydroxyproline colorimentric qauntification according to Stegemann and Stalder (1967). **D)** Neutral sugar composition determined of the molar ratio of the monosaccharides.

ZM: Zygnema - mannitol
ZC: Zygnema - control
ZS: Zygnema - salt
MM: Mesotaenium - mannitol
MC: Mesotaenium - control
MS: Mesotaenium - salt

Supplementary Figure(s) 7. β-Yariv reagent staining

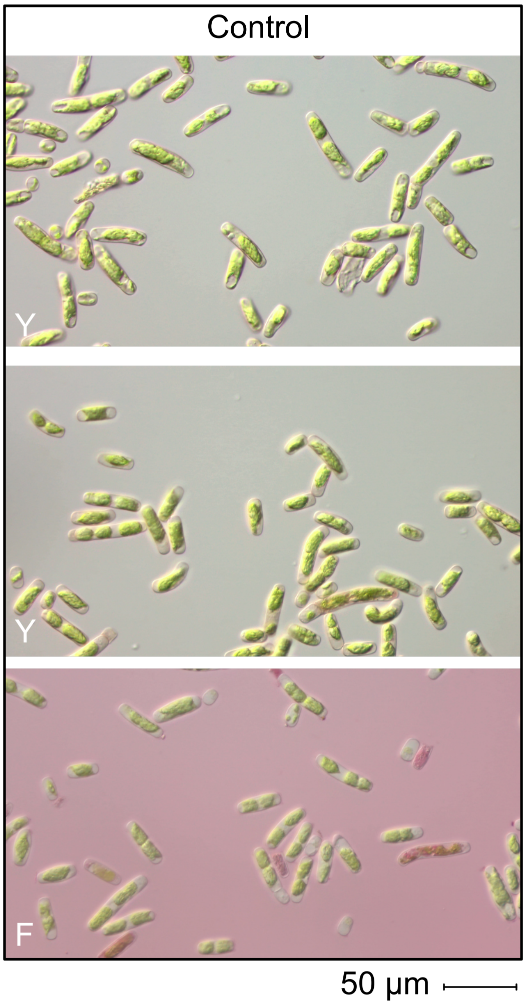

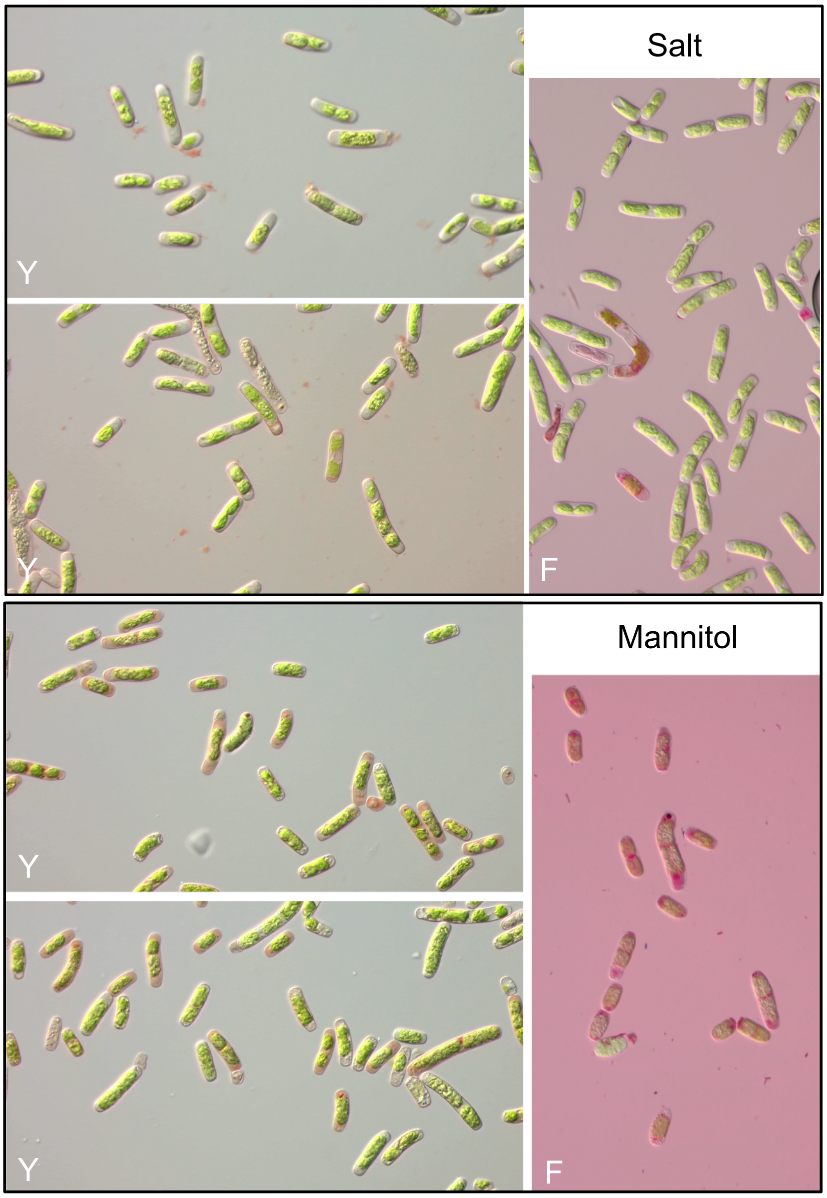

Figure 7A. 200 µM β-yariv (Y) stain or 1% fuchsin (F) with 10 min incubation. Extracellular staining in salt treatments was commonly observed as depicted, ranging from sparse blobs of 1 nm in diameter (bottom) to larger aggregates (top). Such staining was virtually never observed in mannitol treatments, and rarely in control. Staining with 1% fuchsin did not reproduce similar patterns.

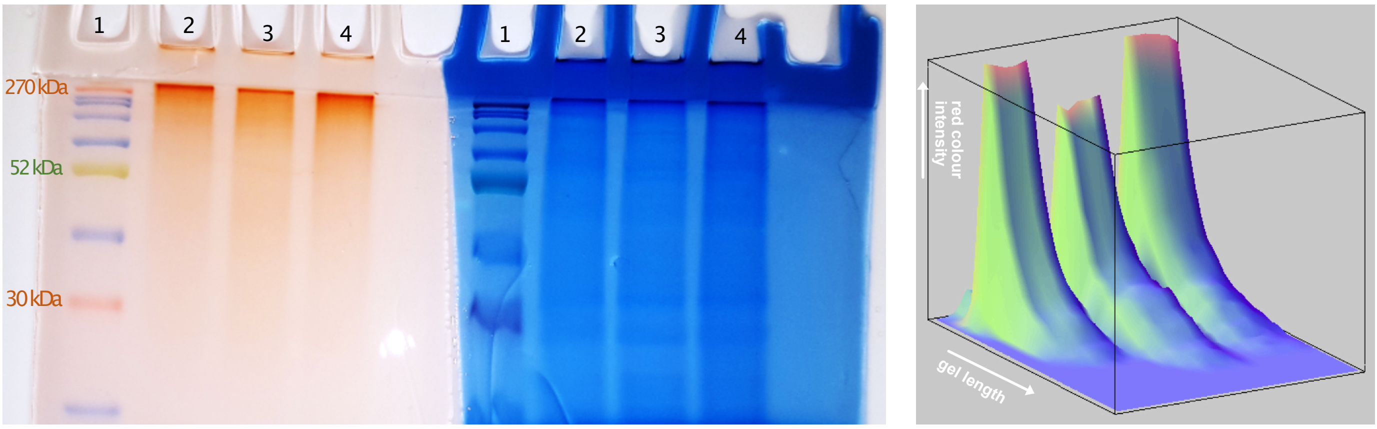

Figure 7B. 12% acrylamide SDS-PAGE gel stained with 0.2% (w/v) ß-yariv reagent on the left side and with Coomassie blue on the right side. Lane 1: abcam prestained protein ladder - extra broad. Lane 2-4: *Mesotaenium* total protein extract, taken from sample after 25 hours of control (2), mannitol (3), or salt (4) treatment. 3D graph on the right show Red channel colour intensities of lanes 2-4 from left to right.

Supplementary Figure(s) 8. Long term NaCl treatment

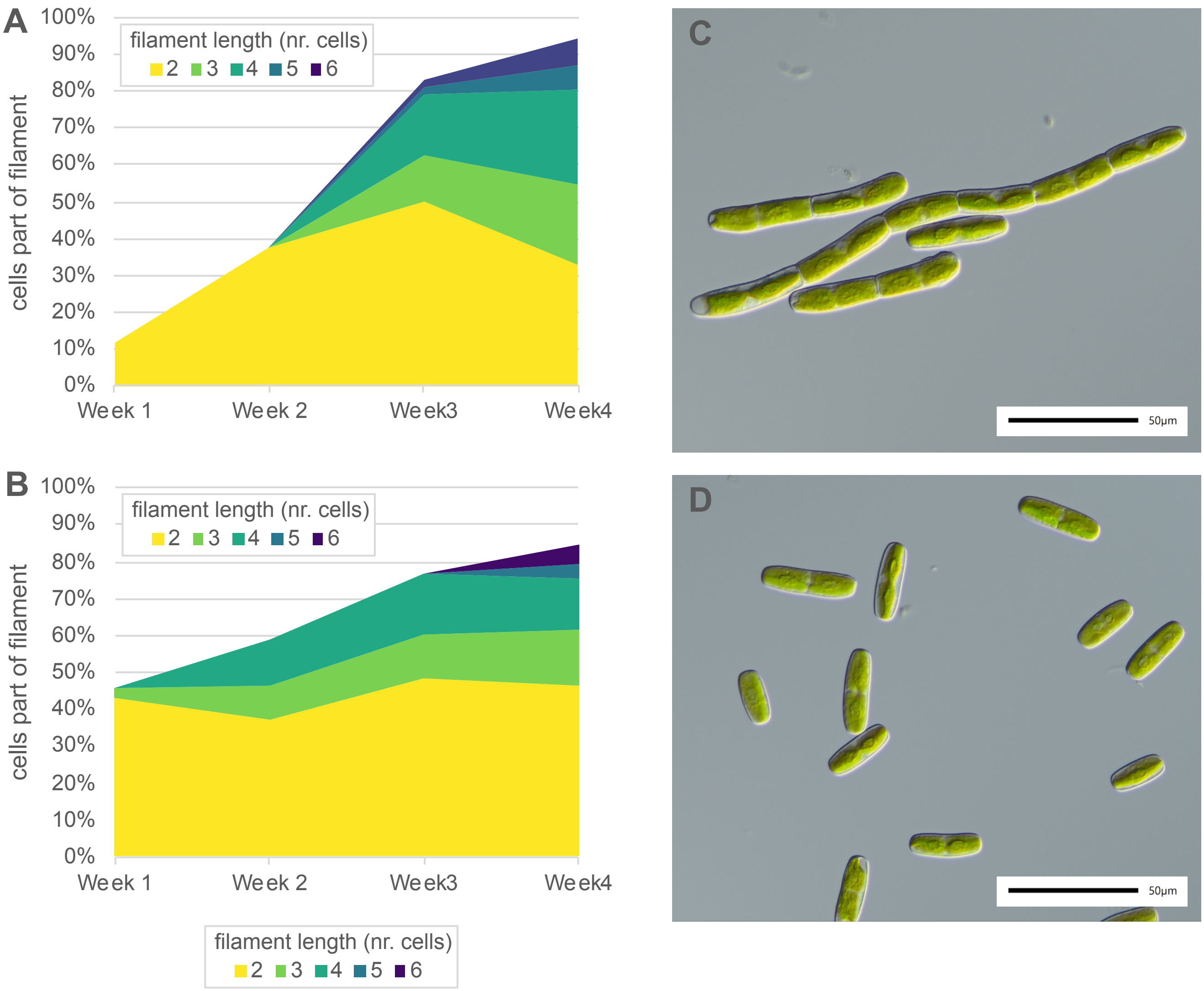

Figure 8A. NaCl-induced short-filamentous growth of *Mesotaenium endlicherianum* SAG12.97. A) Percentage of cells forming filaments after 1-4 weeks of growth on WHM-agar medium supplemented with 0.15 mM NaCl. No filaments (n≥2) were observed in the control. B) Same as panel A, but cells were grown on top of cellophane. C) Light microscopic image of *M. endlicherianum* after 3 weeks of growth in the presence of 0.15 mM NaCl. D) Light microscopic image of the corresponding NaCl-free control from panel C.

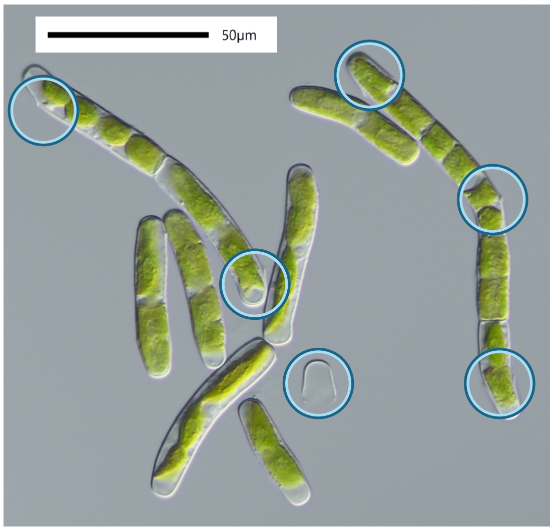

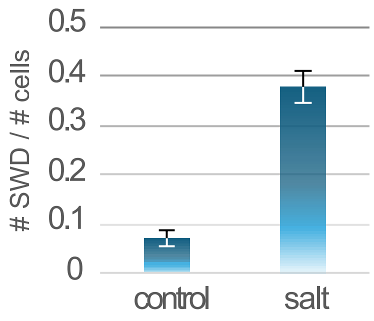

Figure 8B. Secondary wall deposits/features (SWDs) in *M. endlicherianum* after 2 weeks of growth on cellophane foil over agar-solidified WHM medium supplemented with 0.15 M NaCl. Left: Example of SWDs. Top: Ratio of SWDs to cell count for the treatment with NaCl and the control without NaCl.

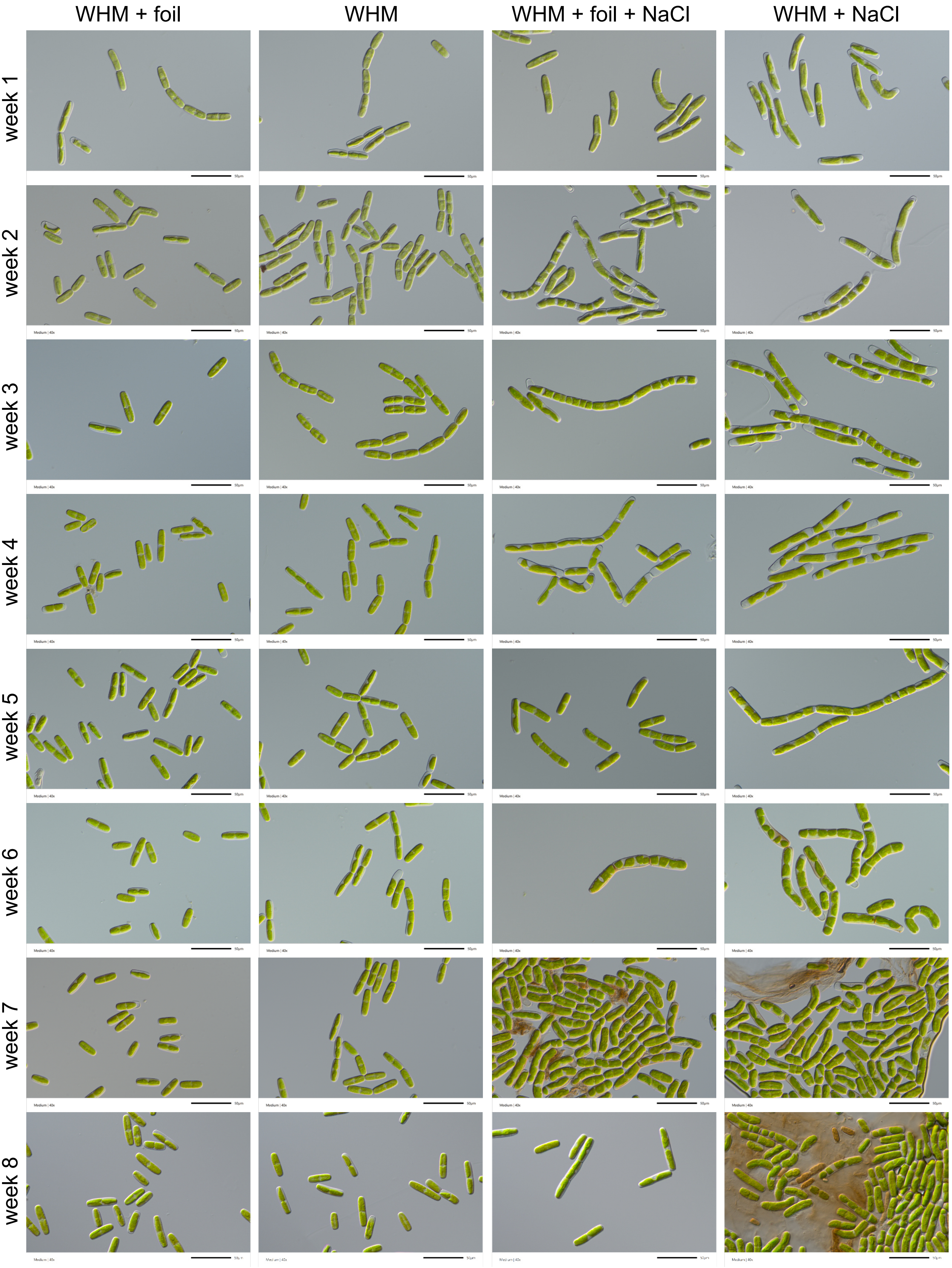

Figure 8C. Microscopic images showing the growth of *M. endlicherianum* over 8 weeks after inoculation onto agar-solidified WHM medium. The images compare growth conditions with and without a cellophane foil overlay, and with and without the addition of 0.15 M NaCl.

Supplementary Figure(s) 9. Protein-RNA analysis workflow

Figure 9. Schematic overview of the data analysis workflow for transcriptomic (yellow boxes), proteomic (red boxes), and combined (orange boxes) datasets. Arrows indicate the flow of output files used as inputs in subsequent steps. 'M' represents the species Mesotaenium, 'Z' denotes Zygnema, and 'B' signifies both species.

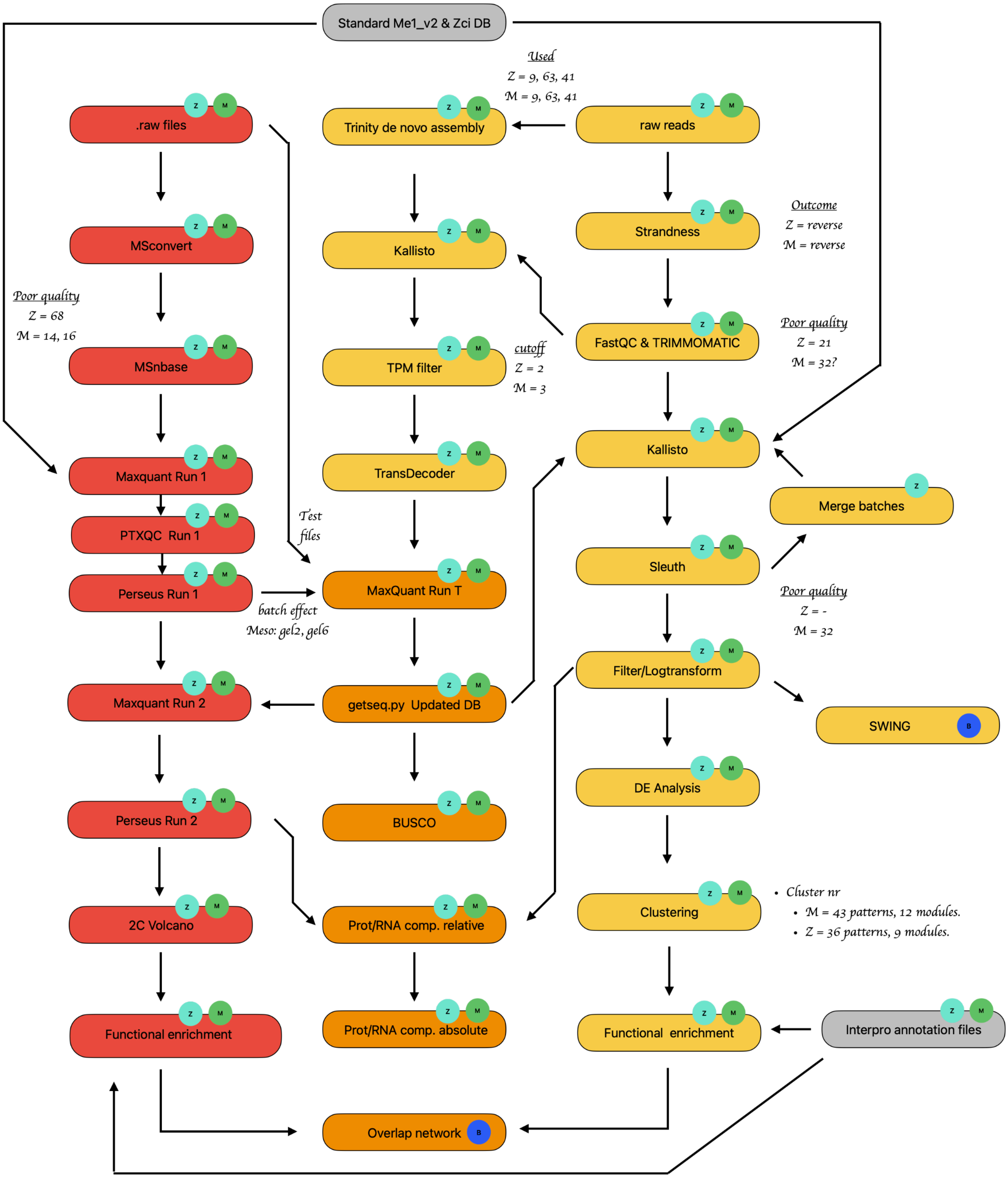

Supplementary Figure(s) 10. transcript patterns

Figure 10. Average temporal transcript patterns calculated from TPM values, clustered by Gaussian mixture modelling. Page 1 (blue module labels): *Zygnema*, page 2 (red module labels): *Mesotaenium*.

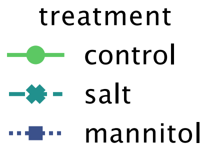

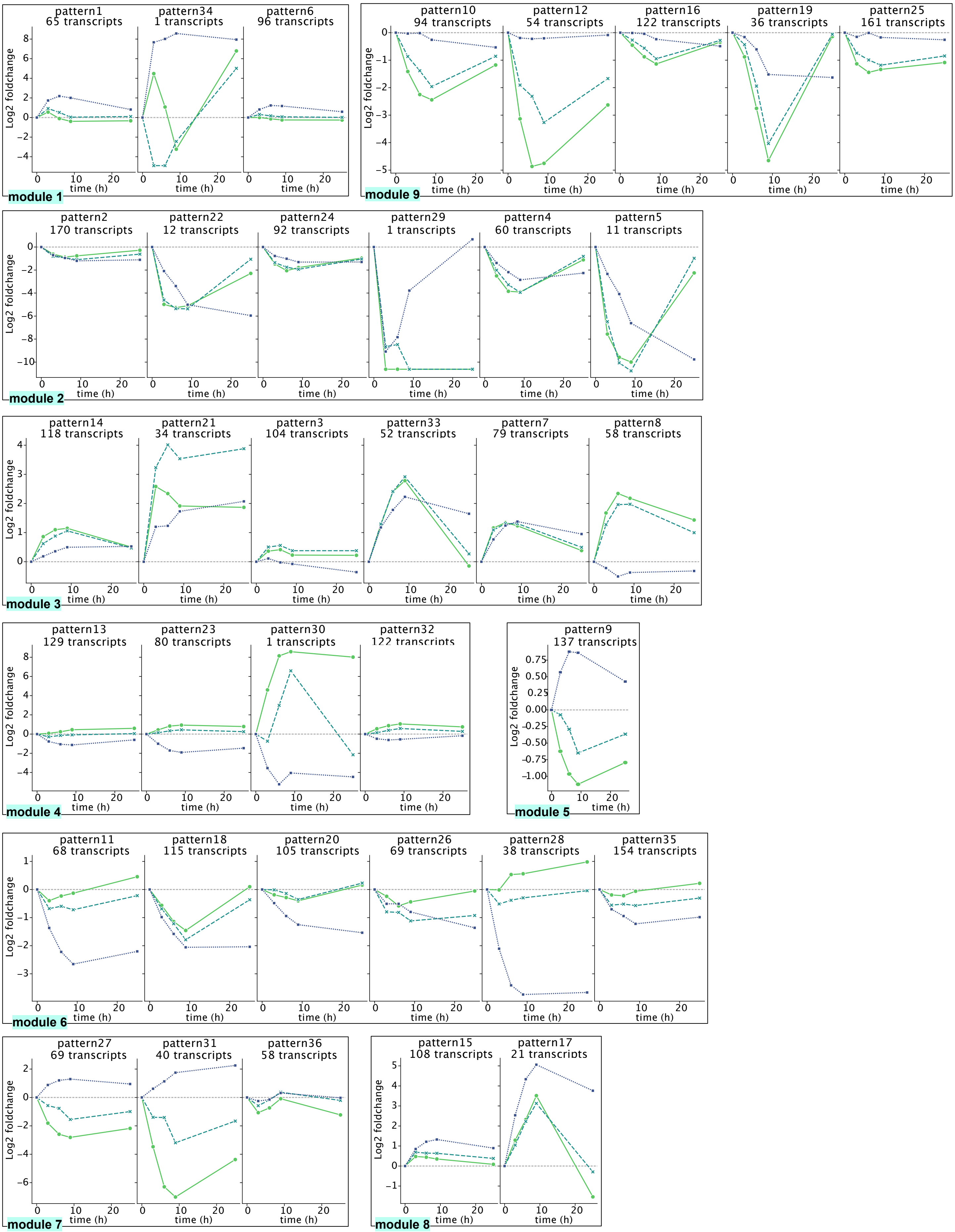

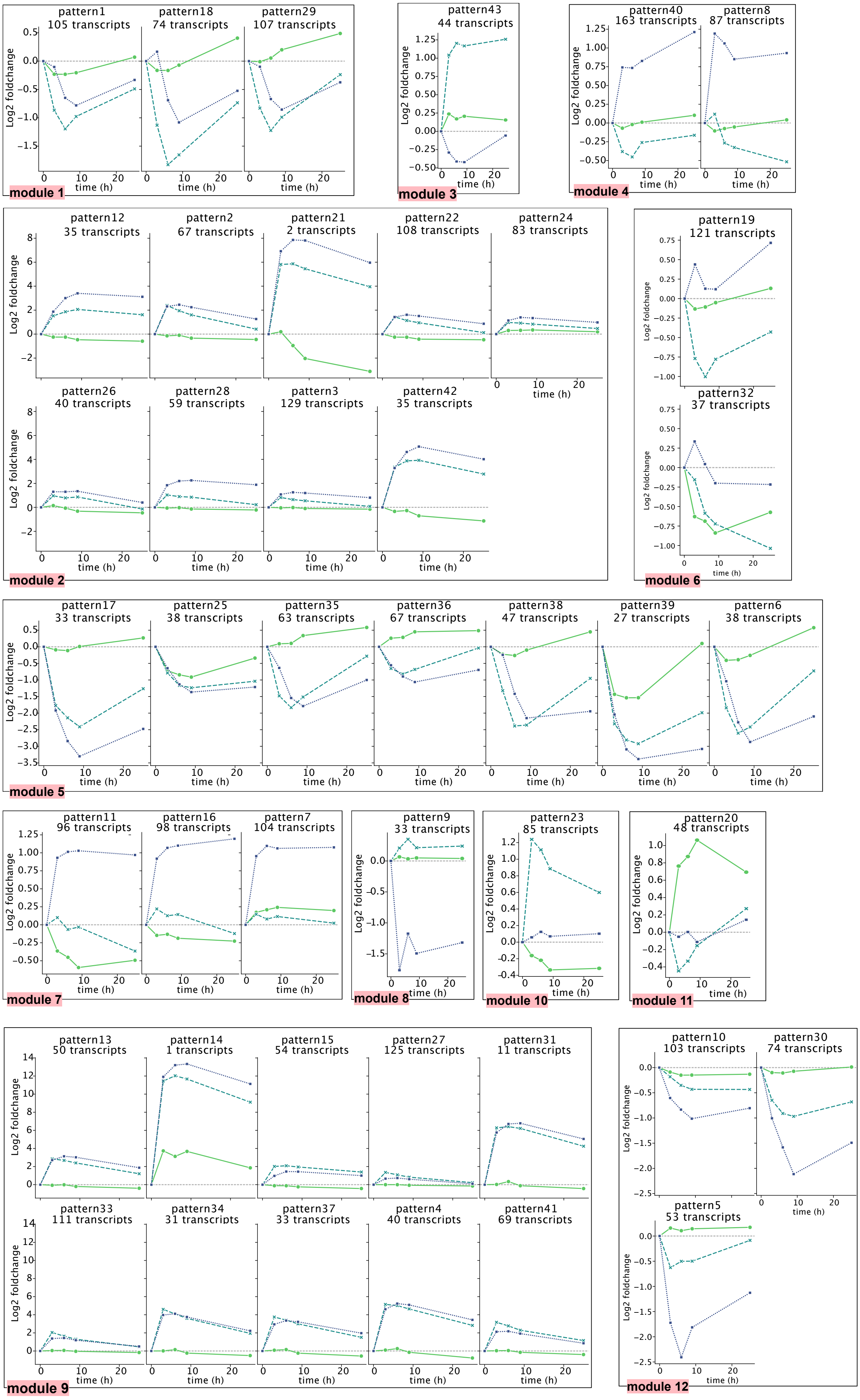

module 1
pattern1
105 transcripts
pattern18
74 transcripts
pattern29
107 transcripts
module 3
pattern43
44 transcripts
module 4
pattern40
163 transcripts
pattern8
87 transcripts
module 2
pattern12
35 transcripts
pattern2
67 transcripts
pattern21
2 transcripts
pattern22
108 transcripts
pattern24
83 transcripts
pattern26
40 transcripts
pattern28
59 transcripts
pattern3
129 transcripts
pattern42
35 transcripts
module 6
pattern19
121 transcripts
pattern32
37 transcripts
module 5
pattern17
33 transcripts
pattern25
38 transcripts
pattern35
63 transcripts
pattern36
67 transcripts
pattern38
47 transcripts
pattern39
27 transcripts
pattern6
38 transcripts
module 7
pattern11
96 transcripts
pattern16
98 transcripts
pattern7
104 transcripts
module 8
pattern9
33 transcripts
module 10
pattern23
85 transcripts
module 11
pattern20
48 transcripts
module 9
pattern13
50 transcripts
pattern14
1 transcripts
pattern15
54 transcripts
pattern27
125 transcripts
pattern31
11 transcripts
pattern33
111 transcripts
pattern34
31 transcripts
pattern37
33 transcripts
pattern4
40 transcripts
pattern41
69 transcripts
module 12
pattern10
103 transcripts
pattern30
74 transcripts
pattern5
53 transcripts
Log2 foldchange
time (h)

Supplementary Figure(s) 11. Full XTH phylogeny

Figure 11. Maximum likelihood phylogeny of XTH homologs detected. Boxes note the subfamily membership within GH16 predicted by dbCAN3.

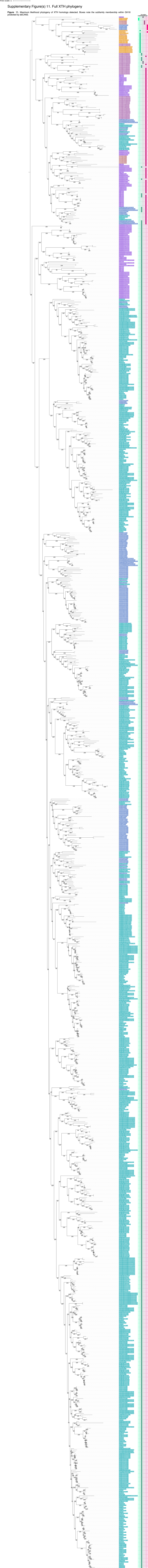

Supplement: Supplementary file 1 — Supplementary Information [file 41467_2026_68329_MOESM1_ESM.pdf]
