# Supplementary material for: Systems acclimation to osmotic stress in zygnematophyte cells
Source: Nat Commun. 2026 Jan 16;17:755. doi: 10.1038/s41467-026-68329-z (PMC12820192; doi:10.1038/s41467-026-68329-z)
Supplement: Supplementary file 4 — Description of Additional Supplementary Files [file 41467_2026_68329_MOESM4_ESM.pdf]

Description of Additional Supplementary Files

Supplementary Data 1: New proteins predicted based on de novo assembled transcripts.

Supplementary Data 2: Differential abundance analysis of all measured protein groups.

Supplementary Data 3: Differential gene expresssion analyses for Mesotaenium (a) and Zygnema (b).

Supplementary Data 4: Metabolite analyses of (a) methanol-based extraction of metabolites after 9h of salt stress measured in postive mode, (b) methanol-based extraction of metabolites after 9h of salt stress measured in both postive and negative mode, (c) methanol-based extraction of metabolites after 3h of salt stress measured in both postive and negative mode, (d) lipid extraction after 9h of salt stress measured in both postive mode, and (e) annotation of the metabolites by biocyc.

Supplementary Data 5: Overview of enriched terms showing overlap between transcript modules and/or protein quadrants from Zygnema and/or Mesotaenium, shared between both (a), unique to Mesotaenium (b), unique to Zygnema (c), and the details on terms and corresponding P values (d, e, f, g).
